# A scoping review of the frequency and clinical presentation of *Taenia solium* taeniasis and human cysticercosis/neurocysticercosis in Papua, Indonesia from 1970 to 2024

**DOI:** 10.1371/journal.pntd.0014702

**Published:** 2026-09-08

**Authors:** Ainrisq A. Rifai, Laura M. Rey, Molly E. Crews, Guilherme G. Verocai, Martial L. Ndeffo-Mbah, Christine M. Budke

**Affiliations:** 1 Department of Veterinary Integrative Biosciences, College of Veterinary Medicine and Biomedical Sciences, Texas A&M University, College Station, Texas, United States of America; 2 Department of Small Animal Clinical Sciences, Clinical and Research Information Service, College of Veterinary Medicine and Biomedical Sciences, Texas A&M University, College Station, Texas, United States of America; 3 Department of Veterinary Pathobiology, College of Veterinary Medicine & Biomedical Sciences, Texas A&M University, College Station, Texas, United States of America; Washington University School of Medicine, UNITED STATES OF AMERICA

## Abstract

**Background:**

*Taenia solium* taeniasis and cysticercosis, including neurocysticercosis (NCC), are neglected tropical diseases disproportionately affecting remote, resource-poor regions. As a leading cause of acquired epilepsy globally, *T. solium* poses a major public health challenge.

**Methodology/principal findings:**

This scoping review evaluated available literature on the prevalence of *T. solium* taeniasis and cysticercosis/NCC, clinical manifestations associated with infection, and diagnostic approaches used in Papua, Indonesia, from 1970 to 2024, following the PRISMA-ScR checklist. From 1,708 identified records, 28 articles underwent data extraction. Pooled prevalence estimates from studies conducted between 2007 and 2015 that used Enzyme-linked Immunoelectrotransfer Blot (EITB) indicated a taeniasis prevalence of 2.0% [95% CI: 1.0% – 6.0%] among household members in Central Papua and 3.0% [95% CI: 1.0% – 10.0%] in Highland Papua households. Furthermore, the pooled prevalence for residents in Central Papua positive by Enzyme-Linked Immunosorbent Assay (ELISA) for cysticercosis (studies from 1969-2015) was 5.0% [95% CI: 2.0% – 13.0%] compared to 29.0% [95% CI: 18.0% – 43.0%] in Highland Papua (studies from 1996-2011). Among 135 patients with headaches and/or seizures from both Central and Highland Papua (studies from 1998-2005), the pooled prevalence for ELISA-positive cysticercosis was 32.0% [95% CI: 9.0% – 69.0%]. Seizures were observed in 19.0% [95% CI: 12.0% – 28.0%] of villagers in Jayawijaya Regency that were ELISA positive for *Taenia solium* cysticercosis (studies from 1996-2001), with 35.0% [95% CI: 9.0% – 75.0%] presenting with subcutaneous nodules. Across all articles, only one study reported the use of brain computed tomography (CT) imaging for confirming NCC, indicating extremely limited access to neuroimaging in the region.

**Conclusions/significance:**

Improving access to gold-standard CT imaging for NCC diagnosis, adopting standardized criteria for NCC diagnosis and management, and targeted public health interventions are urgently needed to reduce the burden of *T. solium* in Papua.

## Author summary

In this study, we reviewed literature on human infection with the pork tapeworm, *Taenia solium,* in the Papua region of Indonesia between 1970 and 2024. This parasite circulates between pigs and humans and can cause serious health issues when the cyst stage infects the brain, resulting in neurocysticercosis. We found that reported infections were most common in Central and Highland Papua provinces, with seroprevalence estimates often exceeding 40% for cysticercosis in community-based studies. However, almost all identified studies used non-gold standard testing methods. These findings reveal a long-term public health problem that has not been fully recognized or addressed by public health policy makers. Future research and health programs should focus on improving testing methods, disease monitoring, and community hygiene and food safety.

## Introduction

*Taenia solium*, commonly known as the pork tapeworm, infects humans through ingestion of undercooked pork, resulting in intestinal infection (taeniasis). Accidental ingestion of *T. solium* eggs due to fecal-oral transmission or autoinfection can result in human infection with the larval stage of the parasite, resulting in cysticercosis. Cysts typically develop in skeletal muscles, but may also affect the lungs, liver, kidneys, and central nervous system [1]. Clinical manifestations associated with cysticercosis depend on the location of the larvae. Cysticerci in the central nervous system can lead to neurocysticercosis (NCC), which can result in various neurological clinical manifestations, including mental and behavioral changes, epilepsy, increased intracranial pressure, and, in severe cases, death [2]. While the diagnosis of NCC is ideally confirmed via computed tomography (CT), serological studies are often used as the main diagnostic tool for *T. solium* cysticercosis in resource-limited areas [3].

NCC is a leading cause of acquired epilepsy worldwide, particularly in low- and middle-income countries (LMICs), and is responsible for an estimated 30% of epilepsy cases in endemic regions [2]. According to the 2021 Global Burden of Disease (GBD) Study, cysticercosis was responsible for an estimated 1,643 (95% confidence interval (CI): 1,113–2,287) deaths and 1.2 million (95% CI: 787,769–1,808,363) Disability Adjusted Life Years (DALYs) lost globally [4]. Despite this burden, taeniasis and cysticercosis remain neglected tropical diseases, disproportionately affecting remote and socioeconomically disadvantaged populations. The transmission of *T. solium* is influenced by multiple factors, including poor hygiene, the absence of meat inspection, and lack of appropriate education about the parasite and its risks [5].

In Southeast Asia, epidemiological data on *T. solium* infections remain limited, particularly in Indonesia. Among the regions of Indonesia, Papua stands out as a potentially significant yet underreported endemic area. The first suspected cases of cysticercosis in Papua, Indonesia were reported in the early 1970s when patients in Central Papua with severe burns were also found to have subcutaneous nodules suggestive of cysticercosis [6]. It was thought that these individuals also had NCC and were falling into fires when they were experiencing NCC-associated epileptic seizures. Between 1977 and 1993, the reported prevalence of cysticercosis diagnosed via Enzyme-linked immunosorbent assay (ELISA) in studies conducted in Central Papua rose from 1.38% to 35.71%, with a concurrent increase in reported cases of epilepsy [7].

In subsequent years, further studies confirmed Papua’s status as an endemic region for *T. solium*. A 2001 study revealed that 56.9% of 65 adults living in *silimos* (a traditional hut-style house) in a village located in Jayawijaya, Highland Papua were seropositive for antibodies against *T. solium* (cysticerci), with 18.5% (12/65) reporting clinical manifestations including seizures, and headaches [8]. Despite such evidence, Papua—and Indonesia as a whole—have been largely excluded from global NCC burden of disease estimates, primarily due to diagnostic limitations such as inconsistent access to CT imaging and the underutilization of standardized case definitions. This omission underscores broader challenges in global health data collection and has likely contributed to underestimating the true burden of NCC globally.

Understanding the epidemiology and clinical presentation of *T. solium* infections is critical for guiding public health interventions in endemic regions. This scoping review aims to synthesize existing literature on the frequency and clinical characteristics of *T. solium* taeniasis and cysticercosis/NCC in Papua, Indonesia, from 1970 to 2024. This information can advise strategies for improving diagnosis, treatment, and control of *T. solium* in Papua.

## Materials and methods

This study follows PRISMA for Scoping Reviews (PRISMA-ScR) checklist (S1 Checklist) and was registered in the Open Science Framework database on May 12, 2025. The protocol can be accessed using the following link: https://doi.org/10.17605/OSF.IO/QHG4K.

### Search strategy and data source

Articles published between January 1, 1970, and December 31, 2024, in English or Indonesian were eligible for inclusion. To qualify, articles had to present original data on the frequency and/or clinical manifestations of presumptive human cases of *T. solium* taeniasis or cysticercosis/NCC, with clearly defined diagnostic methods. While recognizing the importance of porcine cysticercosis for understanding *Taenia solium* transmission, this review was intentionally restricted to human taeniasis and cysticercosis/NCC to characterize the clinical burden and gaps in human case data in Papua.

**Fig 1:**
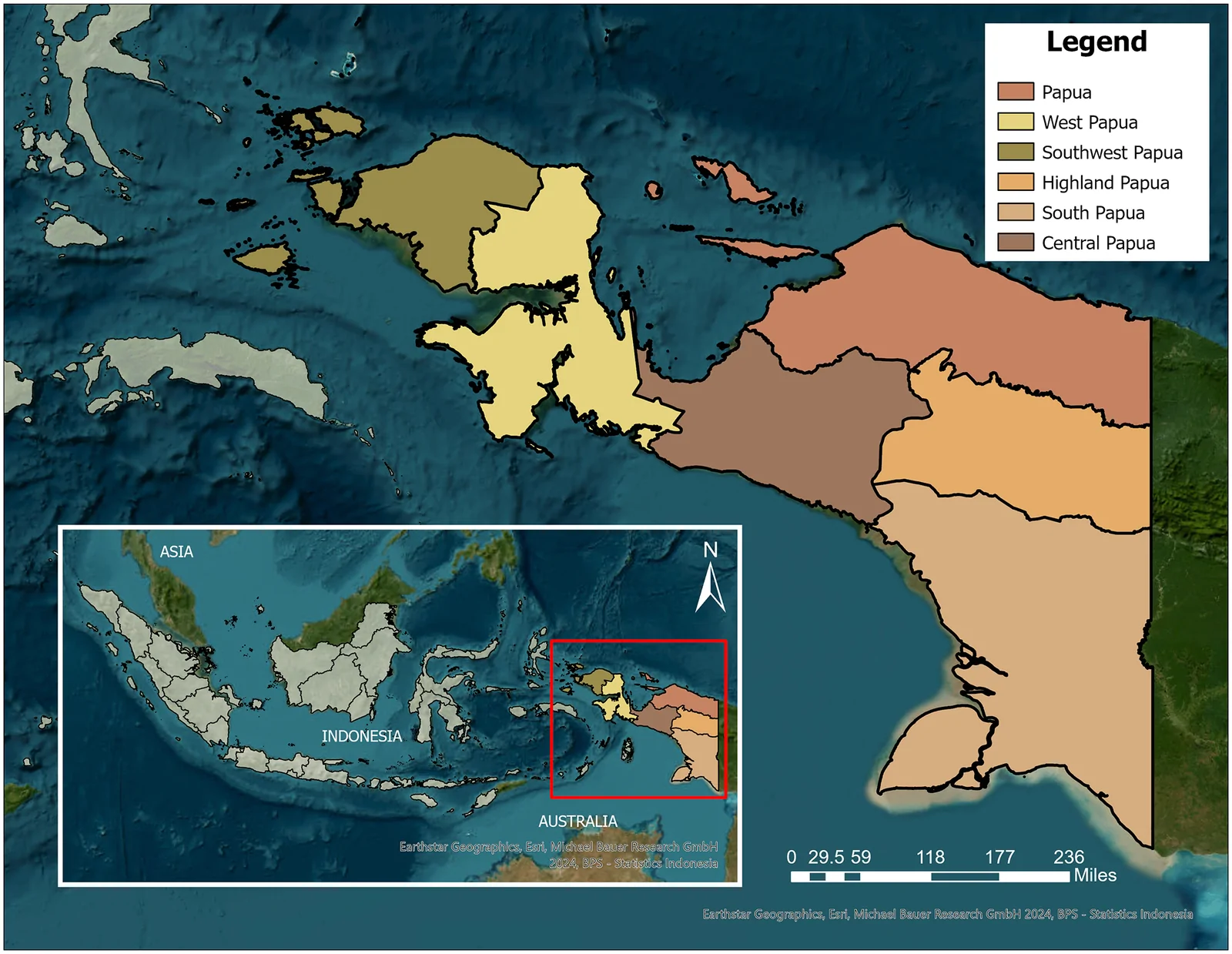
Geographic location of Papua in eastern Indonesia. The map shows Indonesia’s provincial boundaries, with Papua, Indonesia (**red square**) highlighted as the study area. Papua shares a border with the Independent State of Papua New Guinea and represents an endemic region for *T. solium*. Basemap tiles by Esri. Data sources: Esri, DigitalGlobe, GeoEye, i-cubed, USDA FSA, USGS, AEX, Getmapping, Aerogrid, IGN, IGP, swisstopo, and the GIS User Community (https://services.arcgisonline.com/ArcGIS/rest/services/World_Imagery/MapServer).

Acceptable diagnostics approaches for taeniasis included microscopic examination of stool samples and copro-antigen ELISA, and serological techniques such as glycoprotein (GP)-ELISA, immunoblotting, and enzyme-linked immunoelectrotransfer blot (EITB). Diagnosis of cysticercosis/NCC had to involve one or more recognized diagnostic test, including ELISA, immunoblotting, EITB, or Polymerase Chain Reaction (PCR), or cerebrospinal fluid (CSF) analysis. Histopathological examination of cysticerci in subcutaneous nodules, along with diagnostic modalities such as brain imaging, brain biopsy, and autopsy for NCC, were also considered acceptable for inclusion. There were no limitations on study design and sample size. Study area was restricted to Papua, Indonesia. The geographic focus aligned with Indonesia's administrative divisions (UU No. 14–26 Tahun 2022), which divides Papua into six provinces: Papua, Central Papua, Highland Papua, South Papua, Southwest Papua and West Papua (Fig 1).

A systematic search was performed across 12 international databases, including PubMed, Europe PMC, CAB Abstracts, OAIster, and others specializing in science and public health (Table 1). The search terms targeted *Taenia solium* infections using Boolean operators and the following terms: (*Taenia solium* OR *T. solium* OR taeniasis OR taeniosis OR taeniases OR taenosis OR teniasis OR solium OR human neurocysticercosis OR human cysticercosis OR neurocysticercosis OR neurocysticercoses OR cysticercosis OR cysticercoses OR pork tapeworm OR pork tapeworms OR tapeworm OR tapeworms OR cestoda OR cestode OR cestodes) AND (Papua OR Indonesia OR Irian Jaya OR Jayawijaya). Additional manual searches were conducted in Google Scholar, Indonesian repository (NELITI), and World Health Organization's Institutional Repository of Information Sharing (IRIS) for grey literature. Since Google Scholar searches are not reproducible, this search can be considered supplementary, and screening was limited to the first 10 pages due to the large volume of results and diminishing relevance beyond the initial pages. All decisions related to publication sources and search terms were made in consultation with two librarians. All searches were restricted to articles published between January 1, 1970, and December 31, 2024. Search results and execution dates are documented in Table 1.

**Table 1:** Summary of search strategies to evaluate the frequency and clinical characteristics of *T. solium* taeniasis and cysticercosis/NCC in Papua, Indonesia, from 1970 to 2024.

Database	Coverage	Most Recent Search	Number of Records
PubMed	1970 – 2025	12/23/2024 21:04	158
Europe PMC	1970 – 2024	12/23/2024 21:12	74
PMC - NCBI	1970 – 2024	12/23/2024 21:34	17
NLM Catalog	1970 – 2024	12/23/2024 21:40	1
CAB Abstracts via OVID	1970 – 2025	12/23/2024 21:53	364
Global Health via OVID	1970 – 2025	12/23/2024 21:58	244
EMBASE via OVID	1970 – 2025	12/23/2024 22:02	214
Neliti	1970 – 2024	12/22/2024 22:54	25
OAIster	1970 – 2024	12/23/2024 22:20	48
WHO Global Index Medicus	1970 – 2024	12/23/2024 22:25	32
IRIS	–	12/23/2024 22:31	141
Research4Life	01/01/1970 - 12/31/2024	12/23/2024 22:36	188
SciELO	1970 – 2024	12/23/2024 23:12	0
BioMed Central	–	12/23/2024 23:13	67
Google Scholar	01/01/1970 - 12/31/2024	12/23/2024 23:27	105
Handsearching*		02/13/2025 24:45	30
**Total**			**1,708**

*Following Phase II (full-text review), a handsearching process was conducted to identify any additional eligible articles that may not have been captured in the initial search. Relevant references from the included studies were systematically reviewed, and any newly identified articles meeting the inclusion criteria were reassessed and moved to Phase I (title and abstract screening) for further evaluation.

### Data synthesis and analysis

A two-phase screening process was employed using Covidence (SaaS enterprise, Melbourne, Australia) for systematic review management. Covidence was used exclusively for record management, including importing search results, removing duplicates, and organizing the screening process. During Phase I, two independent investigators (AAR and CMB) conducted blinded title and abstract screening to exclude irrelevant articles. Eligible articles provided original data on human *T. solium* infections, reported frequency and/or clinical presentations, and adhered to clear diagnostic definitions. Phase II involved full-text review to exclude articles without full-text availability, original data, or relevant diagnostic and geographic details. Articles with studies focusing solely on animal cases or parasites other than *T. solium* were excluded.

Data extracted in Phase III included study year, language, study design, sample size, geographic location, demographic characteristics, diagnostic methods, clinical manifestations, and frequency of infection. Geographic details such as regency/district, township, and community were included when available. Extracted data were organized in a Microsoft Excel (Microsoft, Washington, USA) spreadsheet. Meta-analyses were conducted for datasets with comparable studies, categorized by study type, diagnostic method, and geographic location. Adjustment of prevalence estimates for diagnostic sensitivity and specificity (e.g., via Bayesian approaches) was not performed. This was due to substantial heterogeneity between identified studies, including differences in diagnostic methods, case definitions, study populations, geographic settings, sampling strategies, and the limited availability of diagnostic test performance data. Pooled proportions were calculated using RStudio IDE (Posit PBC, Massachusetts, USA, version 2024.12.1 + 563, “Kousa Dogwood”) with the inverse variance method, applying logit transformation and the Hartung-Knapp adjustment for random-effects models. Statistical heterogeneity was assessed using I² values, with substantial heterogeneity indicated by values exceeding 50%. Descriptive synthesis of study characteristics and the quantitative reporting of prevalence estimates are provided separately.

### Spatiotemporal analysis for transmission risk

In 2023, the World Health Organization (WHO) released a standardized protocol for mapping *T. solium* endemicity and identifying high-risk areas. As no formal surveillance program has yet classified regions of Papua Island according to this protocol, we applied the WHO criteria to data extracted from articles included in this review to generate spatiotemporal maps of *T. solium* transmission risk in Papua for the period 1970–2025. A binary classification table (Yes/No) was developed to systematically assess the presence or absence of confirmed *T. solium* cases and associated risk factors by region, using the publicly available WHO Risk Classification Tool (Microsoft Excel, Microsoft, Washington, USA). The classification is based on the presence of confirmed human or porcine *T. solium* cases (taeniasis, cysticercosis, or NCC) and two key risk factors: open defecation and backyard pig rearing, both of which are widespread in Papua [9]. Assumptions regarding porcine cysticercosis occurrence in Papua for the purposes of risk classification were informed by prior reviews and reports documenting *T. solium* endemicity and porcine cysticercosis in Indonesia and Papua [10–12], supported by the 2022 global endemicity update [13], while environmental risk factors were informed by WHO WASH data [9] and regional studies on traditional pig farming systems in Papua [14,15]. In settings where neuroimaging is limited, epilepsy is used as a proxy indicator of potential NCC [16]. Risk categories were assigned following a binary decision framework adapted from the WHO, incorporating different combinations of disease evidence (present, absent, or unknown) and risk factors. Risk Areas 1 and 2 correspond to confirmed or strongly suggestive evidence of active transmission (e.g., presence of taeniasis and/or cysticercosis/NCC, with or without porcine data). Risk Areas 3 and 4 represent indications of or potential for active transmission, typically in the absence of confirmed infection but with supporting risk factors and/or proxy indicators (e.g., epilepsy). Risk Areas 5 and 6 reflect lower risk or insufficient evidence of transmission. Using this framework, areas were grouped into three broader categories for mapping: high risk (Risk Areas 1–2), moderate risk (Risk Areas 3–4), and lower risk (Risk Areas 5–6) (S1 Table). All mapping and visualization were conducted using ArcGIS Pro 3.5.0 (Esri, Redlands, California, USA).

## Results

### Literature search

A total of 1,708 records were identified, including 1,407 articles retrieved from 12 databases using database-agnostic search terms, and 271 articles obtained through searching across three websites (NELITI, IRIS, and Google Scholar). Additionally, 30 articles were identified via snowball handsearching of references from studies that passed Phase II and were relevant to the constructed search terms. The Covidence platform identified 793 duplicate records, while 59 duplicates were manually identified during Phase I and Phase II screening.

**Fig 2:**
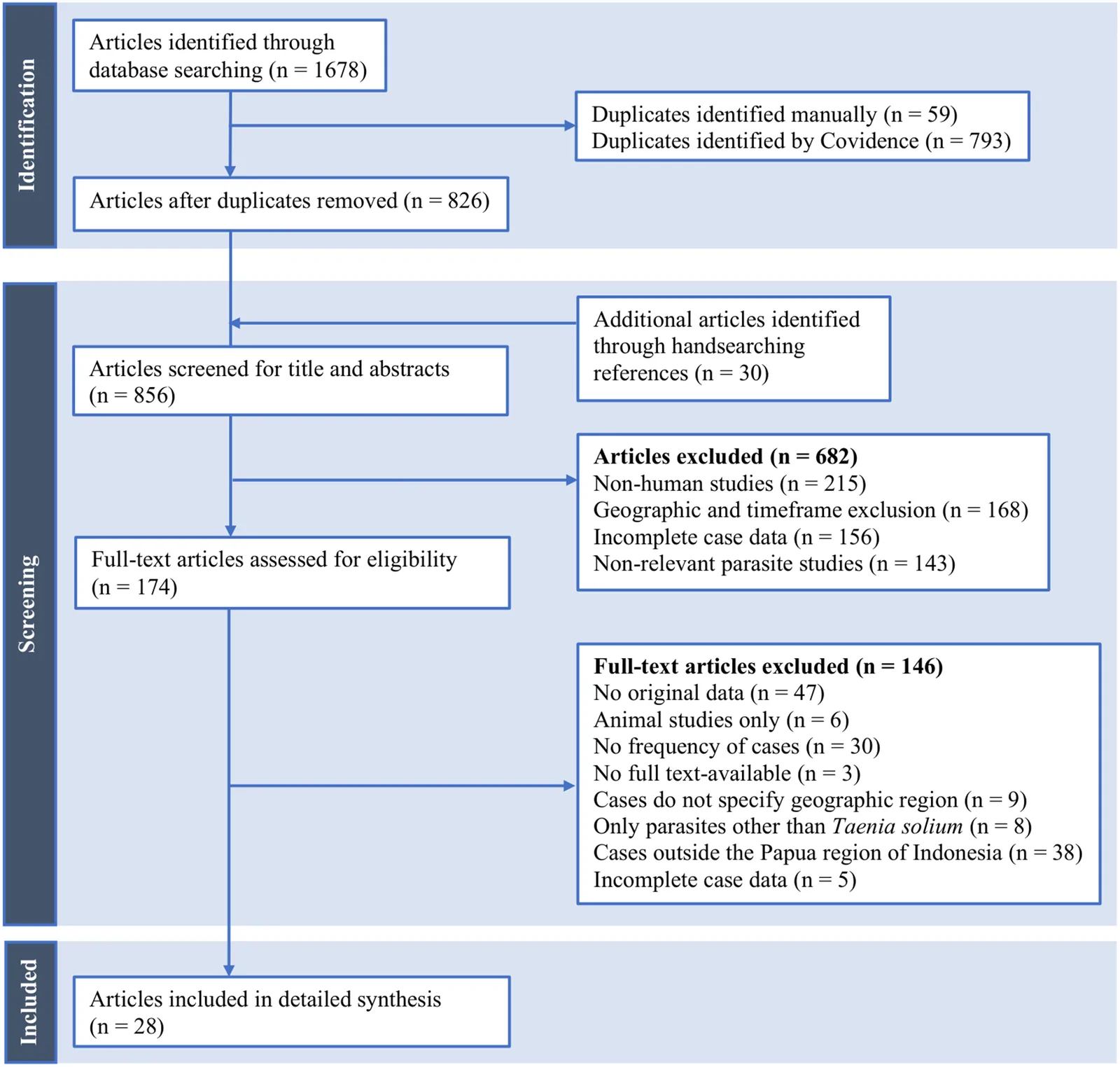
PRISMA-ScR flowchart of the article selection process to evaluate the frequency and clinical presentation of *T. solium* taeniasis and human cysticercosis/NCC in Papua, Indonesia (1970–2024).

The titles and abstracts of 856 articles were evaluated in Phase I. At this stage, 682 articles were excluded, with 174 articles meeting the inclusion criteria to proceed to Phase II for full text review (Fig 1). In Phase II, all eligible articles were uploaded to Covidence for evaluation. From the 174 articles evaluated in Phase II, 146 were excluded, including three lacking accessible full texts. As a result, 28 articles advanced to Phase III for data extraction. The flowchart of the search strategy, following the PRISMA-ScR guidelines, is presented in Fig 2.

### Overview of studies on *T. solium* taeniasis in Papua, Indonesia (1970–2024)

There were 16 included articles examining *T. solium* taeniasis in Papua, Indonesia over the reviewed period. One of the earliest articles, published in 1973, was a case series based on a 1972 study describing the first reported cases of taeniasis in Papua, detected at Enarotali Hospital in Paniai, Central Papua [6]. Central Papua was the most studied region for *T. solium* taeniasis, with 7 of the 16 articles (43.75%) reporting studies from this region. Highland Papua was the second most studied area, represented in 4 articles (25.0%). Two articles reported results from both Central Papua and Highland Papua, while one article reported results from Central Papua, Highland Papua, and Papua province [7,17,18]. One study was conducted at the regional level without identifying the specific province(s) [19]. Among the 16 articles that reported on *T. solium* taeniasis, 4 articles included studies from the 1970s (25%), 1 from the 1980s (6.25%), 2 from the 1990s (12.5%), 4 from the 2000s (25%), and 3 from the 2010s (18.75%). Two articles reported results spanning multiple decades [7,20]. Although only three articles included data for 2010–2019, these studies contained larger populations and numerous study locations [18,21,22].

Two of the 16 taeniasis articles (12.5%) were hospital-based case series, both conducted in Central Papua. These two studies described a total of 56 burn patients presenting to Enarotali Hospital that were taeniasis-positive via microscopic stool analysis and had subcutaneous nodules [23,24]. The remaining 14 taeniasis articles (87.5%) used cross-sectional study designs (12 community-based studies and 2 hospital-based studies). One article included both a community study in the Moni Tribe of Dogondora Valley of West Papua and a hospital-based study in Paniai Regency [25]. Across community-based studies for *T. solium* taeniasis, 14,949 individuals were tested between 1970 and 2019. Central Papua accounted for 43.5% of tested individuals (n = 6,499), followed by Highland Papua with 41.0% of tested individuals (n = 6,123) The number tested was lowest in 1980–1989 (2.9%, n = 434) and highest in 2010–2019 (63.5%, n = 9,495), likely reflecting both increased research activity and the inclusion of larger, more systematically designed studies in recent years.

A total of 857 positive cases of *T. solium* taeniasis were identified in community studies over the review period. Highland Papua accounted for 64.2% of cases (n = 550), while 29.6% of the cases (n = 254) were from Central Papua. The remaining cases were reported from West Papua (5.1%, n = 44), Papua (0.7%, n = 6), and an unspecified location (0.4%; n = 1) [19]. Community-based taeniasis prevalence ranged from as low as 0.0% (0/78) among the Moni tribe in the Dogondora Valley in 1973 (microscopic stool analysis) to as high as 20.4% (39/192) among residents of the Ekari tribe in Paniai in 1987 (ELISA for *T. solium* antibody) [25,26]. Tables 2 and 3 summarize the characteristics of the 28 included articles.

**Table 2:** Summary of included articles on *T.* *solium* taeniasis in Papua, Indonesia (1970–2024).

Reference	Study year(s)	Province(s)	Study Design	Source of Population	Diagnostic Method	Study sample size^a^	Frequency
[24]	1972-1973	Central Papua	Case series	Burns patients with subcutaneous nodules at Enarotali Hospital (16–40-year-olds)	Microscopic stool analysis	13	38.46% (n = 5)
[23]	1973	Central Papua	Case series	Cysticercosis patients with subcutaneous nodules from Enarotali Hospital (>11-year-olds)	Microscopic stool analysis	74	8.11% (n = 6)
[25]	1973	Central Papua	Cross-sectional	Residents from Moni tribe in Dogondora valley	Microscopic stool analysis	78	0.00% (n = 0)
				Patients from Enarotali hospital in Paniai regency		244	8.70% (n = 21)
	1975			Residents from Ekagi tribe in Modio, Dogiyai Regency		82	0.00% (n = 0)
[27]	2000	Central Papua	Cross-sectional	Burns patients at Enarotali Hospital (0–40-year-olds)	Microscopic stool analysis	145	16.55% (n = 24)
[20]	1973	Central Papua	Cross-sectional	Residents from several villages in Enarotali area, Paniai Regency	Microscopic stool analysis	170	10.59% (n = 18)
	1979					350	2.00% (n = 7)
	1994					537	10.43% (n = 56)
[7]	1978	Central Papua	Cross-sectional	Residents with subcutaneous nodules from Ekari tribes in Paniai Regency	Microscopic stool analysis	233	3.00% (n = 7)
	2001			Residents living in 13 *silimos* from Kama villages in Jayawijaya (>14-year-olds)	Copro-Ag ELISA	33	15.15% (n = 5)
[26]	1987	Central Papua	Cross-sectional	Residents from Ekari tribes in Epouto and Waghete villages, Paniai Regency (0–85-year-olds)	Microscopic stool analysis	242	2.89% (n = 7)
					ELISA	192	20.38% (n = 39)
[8]	1993	Highland Papua	Cross-sectional	Residents from Gran Dani Valley village in Jayawijaya Regency (>15-year-olds)	Microscopic stool analysis	380	42.89% (n = 163)
[28]	2000-2001	Highland Papua	Cross-sectional	Residents from Jayawijaya Regency	Copro-Ag ELISA	58	8.62% (n = 5)
[29]	1999	Highland Papua	Cross-sectional	Residents from Jayawijaya Regency	Copro-Ag ELISA	88	15.91% (n = 14)
[30]	2000-2001	Highland Papua	Cross-sectional	Residents from 3 villages in Assologaima area, Jayawijaya Regency	Copro-Ag ELISA	13	0.00% (n = 0)
				Residents from 3 villages in Wamena Kota, Jayawijaya Regency		29	10.34% (n = 3)
[19]	2001	N/A^b^	Cross-sectional	Residents from Irian Jaya	Copro-Ag ELISA	38	7.89% (n = 3)
[17]	2007	Highland Papua	Cross-sectional	Residents from 30 villages in Jayawijaya Regency (6–60-year-olds)	EITB	1253	7.02% (n = 88)
				Residents from 30 villages in Pegunungan Bintang Regency (6–60-year-olds)		391	10.74% (n = 42)
		Central Papua		Residents from 30 villages in Paniai Regency (6–60-year-olds)		633	9.64% (n = 61)
				Residents from 30 villages in Puncak Jaya Regency (6–60-year-olds)		654	1.68% (n = 11)
[21]	2015	Central Papua	Cross-sectional	Residents from Banti village in Mimika Regency (10–46-year-olds)	Microscopic stool analysis	132	0.00% (n = 0)
[18]	2016	Papua	Cross-sectional	Residents from Biak Numfor Regency	EITB	800	0.75% (n = 6)
		Central Papua		Residents from Nabire Regency		798	2.63 = % (n = 21)
				Residents from Deayi Regency		798	0.25 = % (n = 2)
				Residents from Paniai Regency		800	2.38% (n = 19)
				Residents from Intan Jaya Regency		800	0.75% (n = 6)
		Highland Papua		Residents from Nduga Regency		797	6.90% (n = 55)
				Residents from Jayawijaya Regency		799	0.88% (n = 7)
				Residents from Lanny Jaya Regency		800	1.63% (n = 13)
				Residents from Memberamo Tengah Regency		800	16.00% (n = 128)
				Residents from Yalimo Regency		682	3.96% (n = 27)
[22]	2016	West Papua	Cross-sectional	Residents from Teluk Wondama and Pegunungan Arfak Regency	MIA	1489	2.96% (n = 44)

ELISA: Enzyme-linked immunosorbent assay for detection of *T. solium* IgG antibodies.

Copro-Ag ELISA: Coproantigen enzyme-linked immunosorbent assay for *T. solium* antigens.

EITB: Enzyme-linked immunoelectrotransfer blot for *T. solium* antibody detection (rES33 antigen).

MIA: Magnetic immunoassay for *T. solium* taeniasis using rES33 antigen.

^a^number of samples tested from the study population.

^b^data not available.

**Table 3:** Summary of included articles on *T.solium* cysticercosis/NCC in Papua, Indonesia (1970–2024).

Reference	Study year(s)	Province(s)	Study Design	Source of Population	Diagnostic Method	Study sample size^a^	Frequency
[31]	1969-1974	Central Papua	Cross-sectional	Residents of Bilogai village (5–20-year-olds)	Ab-ELISA	166	0.00% (n = 0)
	1969			Residents of Citak village (6–55-years-olds)		130	0.00% (n = 0)
	1974-1977			Residents of Obanu villages (5–50-year-olds)		120	15.83% (n = 19)
	1974			Residents of Bilai village (5–50-year-olds)		76	0.00% (n = 0)
				Residents of Titigi village (5–45-year-olds)		103	0.00% (n = 0)
	1976			Residents of Mek villages (5–60-year-olds)		26	0.00% (n = 0)
	1979			Residents of Asmat village (4–49-year-olds)		145	1.38% (n = 2)
[6]	1972	Central Papua	Case series	A severe burns patient from Ekari tribes at Enarotali Hospital (35-year-olds)	Pathological analysis of the brain (autopsy)	1^d^	100% (n = 1)
[24]	1972-1973	Central Papua	Case series	Burns patients with subcutaneous nodules at Enarotali Hospital (16–40-year-olds)	Histopathology	13	100% (n = 13)
[32]	1992-1995	Highland Papua	Case series	Patients with epileptic seizures from Assologaima Health Center, Jayawijaya Regency	Histopathology	289	4.84% (n = 14)
[20]	1973	Central Papua	Cross-sectional	Residents from several villages in Enarotali area, Paniai Regency	Ab-ELISA	170	25.29 (n = 43)
						74	28.38% (n = 21)
	1979					350	17.43% (n = 61)
	1994					537	21.60% (n = 116)
	2000	Highland Papua		Residents from several villages in Baliem Valley, Jayawijaya Regency		70	60.00% (n = 42)
[33]	1974-1977	Central Papua	Case series	Cysticercosis patients from Enarotali Hospital (13–46-year-olds)	Ab-ELISA	31	61.29% (n = 19)
[34]	1977	Central Papua	Cross-sectional	Residents from Obanu village in Paniai Regency (0–50-year-olds)	CIEP	125	41.60% (n = 52)
[7]	1998	Highland Papua	Cross-sectional	Residents with epileptic seizures from Jayawijaya	Ab-ELISA and/or IB^c^	41	19.51% (n = 8)
				Residents with epileptic seizures and subcutaneous nodules from Jayawijaya		12	100.00% (n = 12)
				Residents with subcutaneous nodules from Jayawijaya		18	55.56% (n = 10)
				Residents with taeniasis from Jayawijaya		16	37.50% (n = 6)
				Healthy residents from Jayawijaya		68	23.53% (n = 16)
	1999			Residents with subcutaneous nodules from Wamena Kota and Assologaima		7	57.14% (n = 4)
				Residents with epileptic seizures from Wamena Kota and Assologaima		27	62.96% (n = 17)
				Residents with epileptic seizures and subcutaneous nodules from Wamena Kota and Assologaima		9	55.56% (n = 5)
				Healthy residents from Wamena Kota and Assologaima		39	48.72% (n = 19)
	2001			Residents living in 13 *silimos* from Kama villages in Jayawijaya (>14-year-olds)		65	56.92% (n = 37)
[35]	1986	Central Papua	Cross-sectional	Refugee from Paniai Regency in Papua New Guinea (0–90-year-olds)	IB	79	1.27% (n = 1)
[36]	1991-1995	Highland Papua	Cross-sectional	Residents with epileptic seizures and headaches from Jayawijaya	IB	18	66.67% (n = 12)
				Residents with subcutaneous nodules from Jayawijaya		31	64.52% (n = 20)
[28]	1996	Highland Papua	Cross-sectional	Residents from Assologaima area in Jayawijaya Regency (18–45-year-olds)	Ab-ELISA	17	70.59% (n = 12)
						32	62.50% (n = 20)
						47	25.53% (n = 12)
					Histopathology	96	14.58% (n = 14)
	1997				Ab-ELISA		47.92% (n = 46)
[29]	1996	Highland Papua	Cross-sectional	Residents from Assologaima area in Jayawijaya Regency	Ab-ELISA	96	45.83% (n = 44)
	1998-2001			Residents from Hubikosi area in Jayawijaya Regency		43	18.60% (n = 8)
	1998-2002			Residents from Wamena Kota area in Jayawijaya Regency		632	15.66% (n = 99)
	1998			Residents from Kurulu area in Jayawijaya Regency		131	39.69% (n = 52)
	1997	South Papua		Residents from Merauke Regency		30	3.33% (n = 1)
	1998					60	0.00% (n = 0)
	2003-2004	West Papua		Residents from Anggi area in Manokwari Regency		177	2.26% (n = 4)
	2003			Residents from Minyambow area in Manokwari Regency		97	4.12% (n = 4)
	2004-2005	Central Papua		Residents from Monamani area in Nabire Regency		54	9.26% (n = 5)
	2004			Residents from Idakebo area in Nabire Regency		14	35.71% (n = 5)
				Residents from Nabire area in Nabire Regency		37	0.00% (n = 0)
				Residents from villages in Paniai Regency		61	1.64% (n = 1)
[37]	1997	Highland Papua	Cross-sectional	Refugee from Jayawijaya in Papua New Guinea	Ab-ELISA and IB^c^	541	2.96% (n = 16)
	1999			Pig owners and their relatives/neighbors with Taeniasis from Jayawijaya Regency	Ab-ELISA	57	63.16% (n = 36)
				Healthy pig owners and their relatives/neighbors from Jayawijaya Regency		103	43.69% (n = 45)
				Residents from Jayawijaya with taeniasis		14	50.00% (n = 7)
[38]	1999	Highland Papua	Cross-sectional	Healthy pig owners and their relatives/neighbors from Jayawijaya Regency	IB	160	50.63% (n = 81)
[30]	2000-2001	Highland Papua	Cross-sectional	Residents from 3 villages in Assologaima area, Jayawijaya Regency	IB	51	35.29% (n = 18)
				Residents from 3 villages in Wamena Kota, Jayawijaya Regency		38	68.42% (n = 26)
[39]	2001	Highland Papua	Cross-sectional	Residents from Kama village in Jayawijaya Regency (15–65-year-olds)	Ab-ELISA and IB^c^	28	32.14% (n = 9)
[17]	2007	Highland Papua	Cross-sectional	Residents from 30 villages in Jayawijaya Regency (6–60-year-olds)	EITB	1253	20.83% (n = 261)
				Residents from 30 villages in Pegunungan Bintang Regency (6–60-year-olds)		391	2.56% (n = 10)
		Central Papua		Residents from 30 villages in Paniai Regency (6–60-year-olds)		633	29.23% (n = 185)
				Residents from 30 villages in Puncak Jaya Regency (6–60-year-olds)		654	1.99% (n = 13)
[40]	2011	Highland Papua	Cross-sectional	Residents from Jayawijaya	Ab-ELISA and/or IB^c^	181	15.47% (n = 28)
	2012					109	8.26% (n = 9)
[41]	2011	Highland Papua	Cross-sectional	Residents from Wamena Kota, Jayawijaya Regency	Ab-ELISA	286	20.98% (n = 60)
[21]	2015	Central Papua	Cross-sectional	Residents from Banti village in Mimika Regency (10–46-year-olds)	Ab-ELISA	131	0.76% (n = 1)
					Brain CT	3^d^	100.0% (n = 3)
[22]	2016	West Papua	Cross-sectional	Residents from Teluk Wondama and Pegunungan Arfak Regency	MIA	1489	3.16% (n = 47)

ELISA: Enzyme-linked immunosorbent assay for detection of *T. solium* larvae IgG antibodies.

CIEP: Counter immunoelectrophoresis precipitin test for *T. solium larvae* antigen-antibody complexes.

IB: Immunoblotting for cysticercosis.

EITB: Enzyme-linked immunoelectrotransfer blot for cysticercosis antibody detection (rT24H antigen).

Brain CT: Brain computed tomography scan used to diagnose neurocysticercosis.

MIA: Magnetic immunoassay for *T. solium* cysticercosis using rT24H antigen.

^a^Number of samples tested from the study population.

^b^Data not available.

^c^Where multiple diagnostic tests are reported (“and/or”), the original studies did not specify whether tests were used in parallel, sequentially, or for confirmatory purposes. ^d^Confirmed cases of neurocysticercosis.

### Overview of studies on *T. solium* cysticercosis/NCC in Papua, Indonesia (1970–2024)

Twenty-two articles were identified that reported the prevalence of *T. solium* cysticercosis/NCC, with two articles specifically evaluating NCC via imaging or at autopsy. Among the 20 studies not specific to NCC, 4 (20%) were conducted in the 1970s, 1 (5%) in the 1980s, 5 (25%) in the 1990s, 4 (20%) in the 2000s, and 4 (20%) in the 2010s. Three articles reported results spanning multiple decades [7,20,29]. With regards to geographic distribution, 30.0% (n = 6) of the articles included data from Central Papua, 50.0% (n = 10) from Highland Papua, and 5.0% (n = 1) from West Papua. Two articles (10%) presented data from both Central and Highland Papua, while one article reported data from Highland Papua, South Papua, West Papua, and Central Papua [17,20,29].

Of the 20 studies not focusing on NCC, most (85.0%, n = 17) were community-based cross-sectional designs, while three studies (15.0%) were hospital-based case series. Community-based studies collectively examined 10,361 individuals. The largest proportion of evaluated individuals came from Highland Papua (46.5%, n = 4,823), followed by Central Papua (35.6%, n = 3,685). Among these community studies, 1,702 seropositive individuals were identified, with most from Highland Papua (65.9%, n = 1,121) and Central Papua (30.8%, n = 525). The largest number of seropositive individuals were reported in 1990–1999 (43.4%, n = 738), while only one seropositive person was reported in 1980–1989 (0.1%). Hospital-based case series examined 333 patients overall, with 13.2% (n = 44) from hospitals in Central Papua during 1970–1979, and 86.8% (n = 289) from hospitals and health centers in Highland Papua during 1990–1999. A total of 46 positive cases were reported in hospital-based studies, including 32 patients (69.6%) from Central Papua and 14 patients (30.4%) from Highland Papua.

Of the two NCC-specific studies, one reported NCC in a 35-year-old male with severe burns from Central Papua. The diagnosis of NCC was made postmortem through autopsy [6]. Forty-three years later, a community study among residents aged 10–46-years in a village located in Mimika Regency of Central Papua identified three cases of NCC using brain CT [21]. Village residents (n = 132) were first screened for seizures, with three individuals with seizures identified. These three individuals were referred to the nearest hospital for brain and leg CT imaging, which revealed the presence of *T. solium* cysts in their brains [21].

### Diagnostic approaches for *T. solium* taeniasis and human cysticercosis/NCC in Papua, Indonesia (1970–2024)

**Fig 3:**
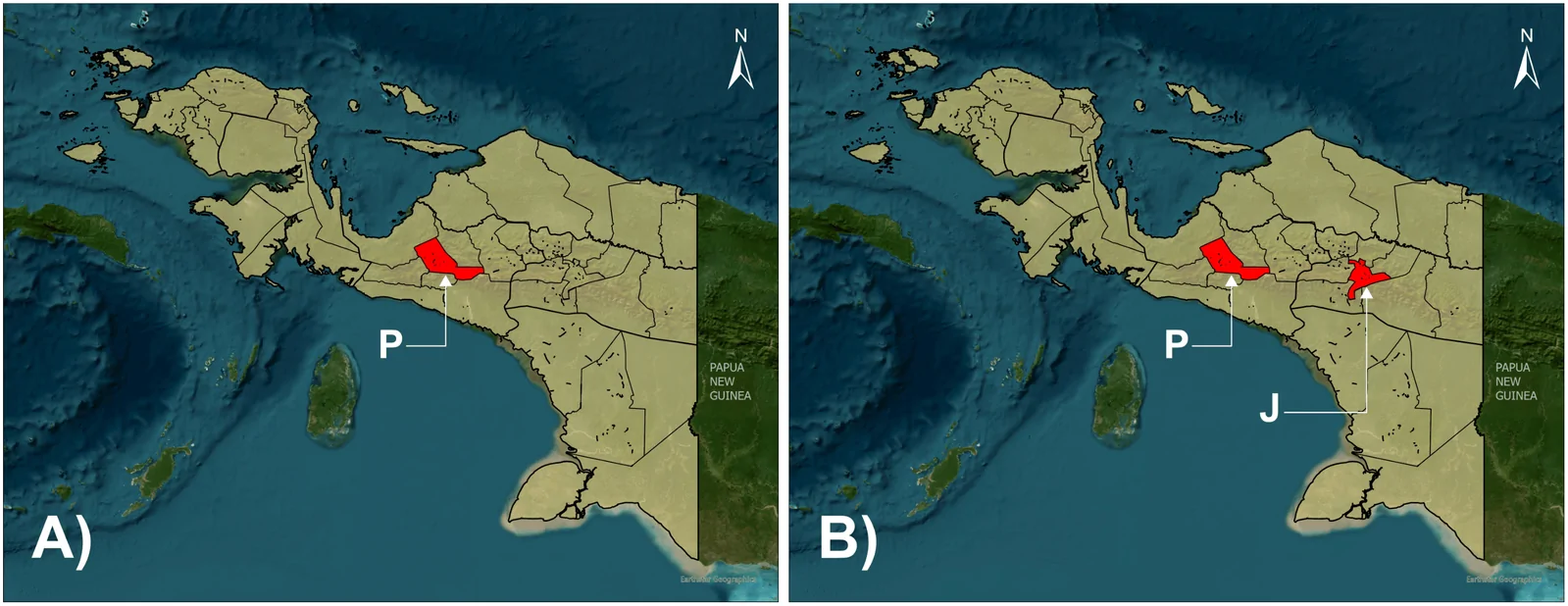
Spatial distribution of the cumulative count of reported positive *T. solium* taeniasis cases identified by microscopic stool analysis across regions of Papua. A. 1970–1979: Distribution of reported taeniasis cases in Paniai Regency (**P**; n = 88) in Central Papua Province. B. 1990–1999: Distribution of reported taeniasis cases in Paniai Regency (**P**; n = 56) in Central Papua and Jayawijaya Regency (**J**; n = 163) in Highland Papua. Basemap tiles by Esri. Data sources: Esri, DigitalGlobe, GeoEye, i-cubed, USDA FSA, USGS, AEX, Getmapping, Aerogrid, IGN, IGP, swisstopo, and the GIS User Community (https://services.arcgisonline.com/ArcGIS/rest/services/World_Imagery/MapServer).

Among the 16 included taeniasis articles (Table 2), nine (31.6%) employed microscopic analysis of stool samples [23,24]. One article specifically mentioned the use of the Kato-Katz technique, while most provided limited detail regarding their microscopic methods [21]. Microscopic analysis was predominantly used in the earlier phases (pre-1995) of research, with the most recent application in 2015 [21]. Other diagnostic methods for taeniasis included EITB copro-antigen ELISA, ELISA for detection of IgG antibodies against *T. solium* in human serum, and magnetic immunoassay [7,17,18,22,26,28–30,37]. The spatial distribution of reported positive *T. solium* taeniasis cases identified by microscopic analysis of stool across Papua is presented in Fig 3. No taeniasis cases based on microscopic analysis of stool samples were reported in the literature from 1980-1989 or after 1999.

**Fig 4:**
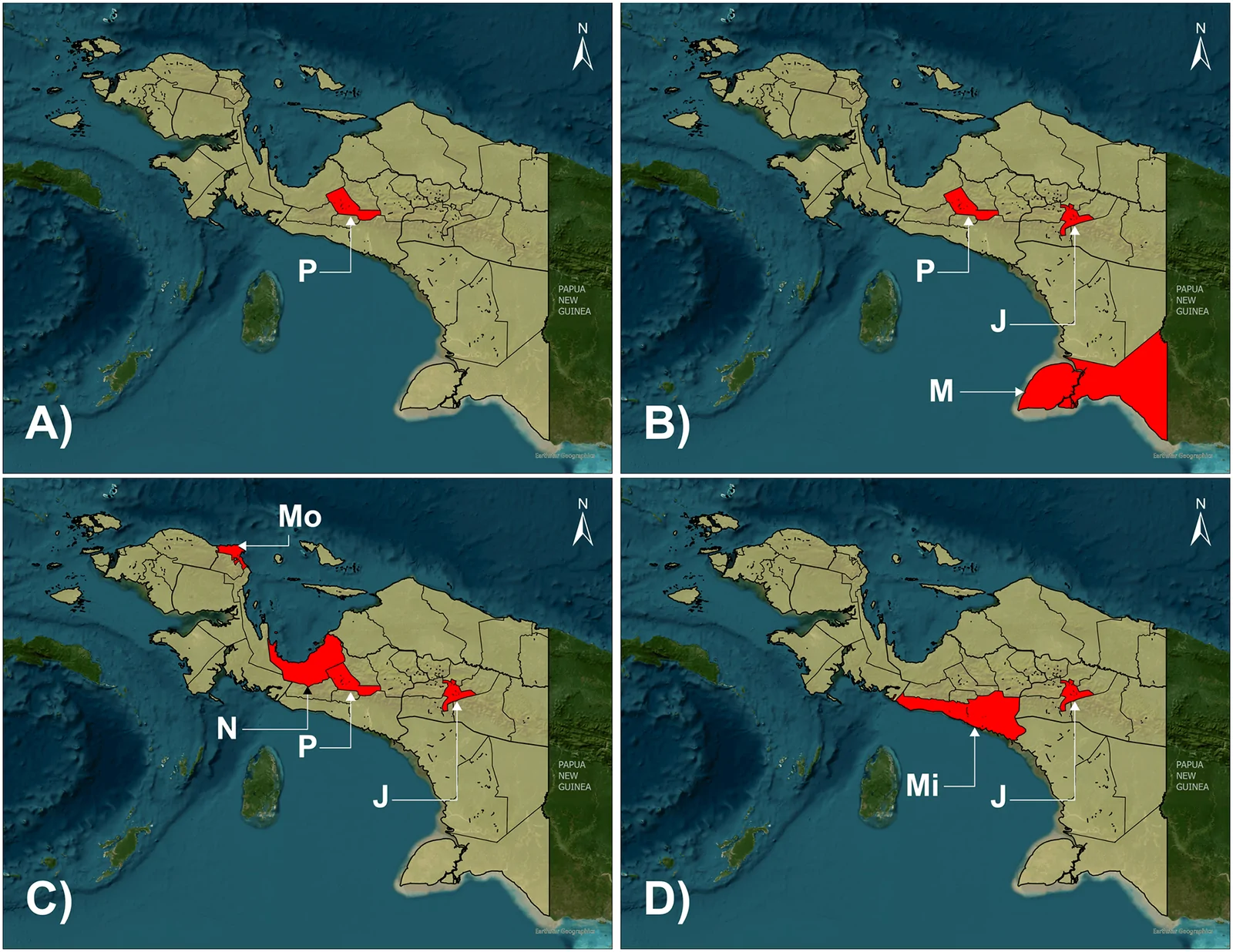
Spatial distribution of the cumulative count of reported seropositive human *T. solium* cysticercosis cases identified by ELISA across regions of Papua. A. 1970–1979: Reported *T. solium* cysticercosis cases in Paniai Regency (**P**; n = 165) in Central Papua Province. B. 1990–1999: Reported *T. solium* cysticercosis cases in Merauke Regency (**M**; n = 1) in South Papua, Paniai Regency (**P**; n = 116) in Central Papua, and Jayawijaya Regency (**J**; n = 494) in Highland Papua. C. 2000–2009: Reported *T. solium* cysticercosis cases in Paniai Regency (**P**; n = 1) in Central Papua, Manokwari Regency (**Mo**; n = 8) in West Papua, Nabire Regency (**N**; n = 10) in Central Papua and Jayawijaya Regency (**J**; n = 88) in Highland Papua. D. 2010–2019: Reported *T. solium* cysticercosis cases in Mimika Regency (**Mi**; n = 1) in Central Papua and Jayawijaya Regency (**J**; n = 97) in Highland Papua. Basemap tiles by Esri. Data sources: Esri, DigitalGlobe, GeoEye, i-cubed, USDA FSA, USGS, AEX, Getmapping, Aerogrid, IGN, IGP, swisstopo, and the GIS User Community (https://services.arcgisonline.com/ArcGIS/rest/services/World_Imagery/MapServer).

Diagnostic methods for cysticercosis in Papua have predominantly consisted of serological testing, with Ab-ELISA for detecting antibodies against the parasite most used (Fig 4). Eight of the 20 included articles reporting presumed cysticercosis cases used Ab-ELISA as the sole diagnostic method [19–21,28,29,31,33,41]. Ab-ELISA was also frequently combined with immunoblotting: two articles explicitly described employing both methods together [19,39], while another two articles indicated using Ab-ELISA and/or immunoblotting in their diagnostic protocols [7,40]. Four of the 20 articles utilized immunoblotting alone for cysticercosis diagnosis [30,35,36,38]. While serology remained the dominant approach, histological analysis of biopsied subcutaneous nodules was also used, particularly in earlier studies (1972–1996) [24,28,32]. More recently introduced diagnostic methods included Counter-Immunoelectrophoresis (CIEP) testing [34], and a magnetic particle immunoassay using the cysticercosis antigen rT24H. The latter was applied in West Papua in 2016, yielding a reported cysticercosis frequency of 3.16% [22].

### *T. solium* taeniasis prevalence in Papua, Indonesia (1970–2024)

**Fig 5:**
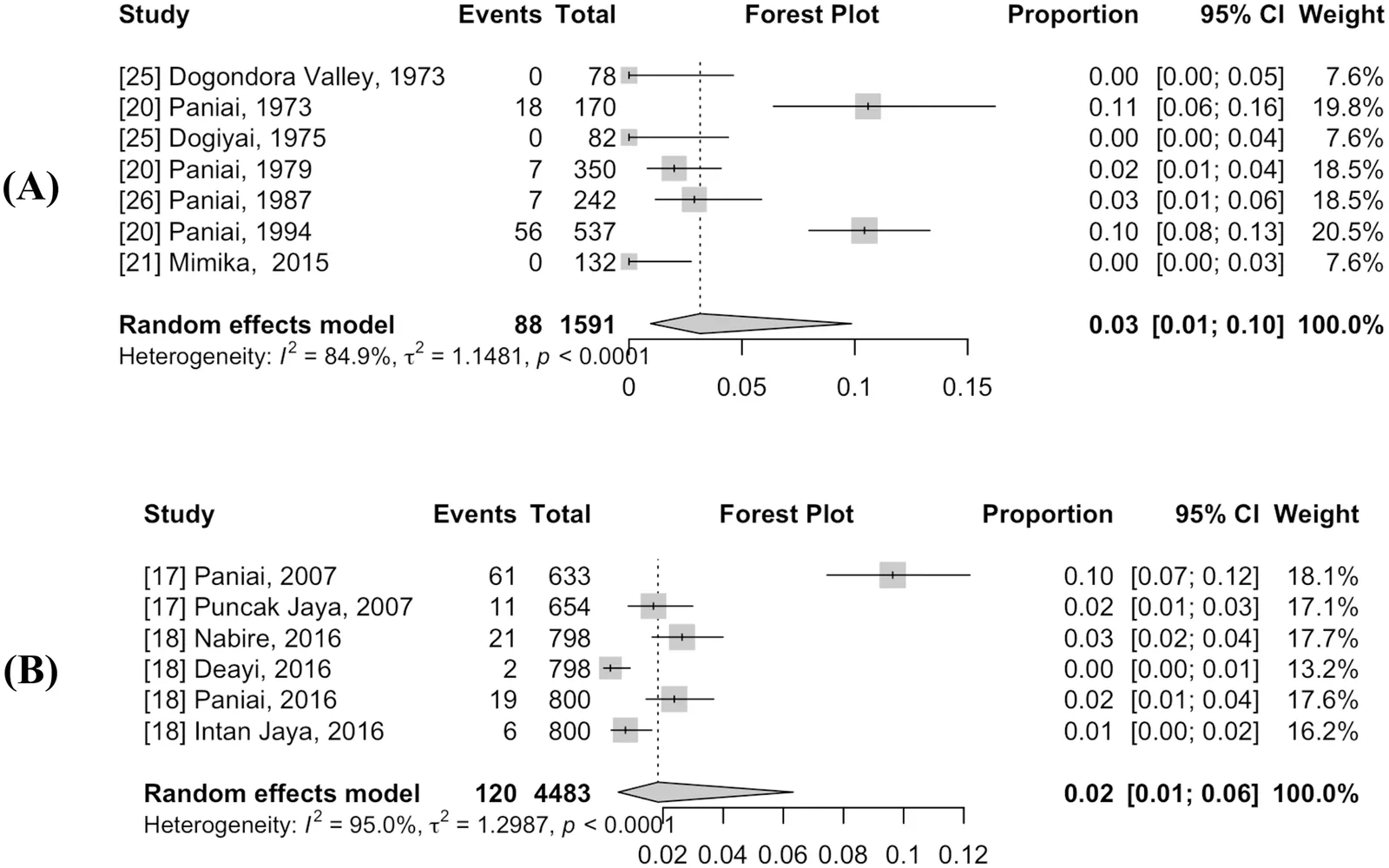
Pooled prevalence of *T. solium* taeniasis in Central Papua. The forest plots represent taeniasis prevalence from community-based studies in Central Papua using A) microscopic stool analysis and B) recombinant rES33 Enzyme-linked immunotransfer blot (EITB).

Pooled prevalence estimates for *T. solium* taeniasis were produced when there were at least three study populations in a region evaluated using the same study design and diagnostic method. For Central Papua (Fig 5), the pooled prevalence of *T. solium* taeniasis using microscopic stool analysis in community-based studies was 3.0% (95% CI: 1.0%–10.0%, I^2^: 84.9%) [8,20,21,25,26]. It should be noted that four of the five included data points were from the Paniai Regency, with sampling dates representing 1973, 1979, 1987, and 1994 [20,26]. In contrast, pooled prevalence in Central Papua, based on EITB with recombinant antibody rES33 in community-based samples, was 2.0% (95% CI: 1.0%–6.0%, I^2^: 95.0%) [17,18]. Two of the included data points for this analysis were also from Paniai Regency, representing the years 2007 and 2016 [17,18].

**Fig 6:**
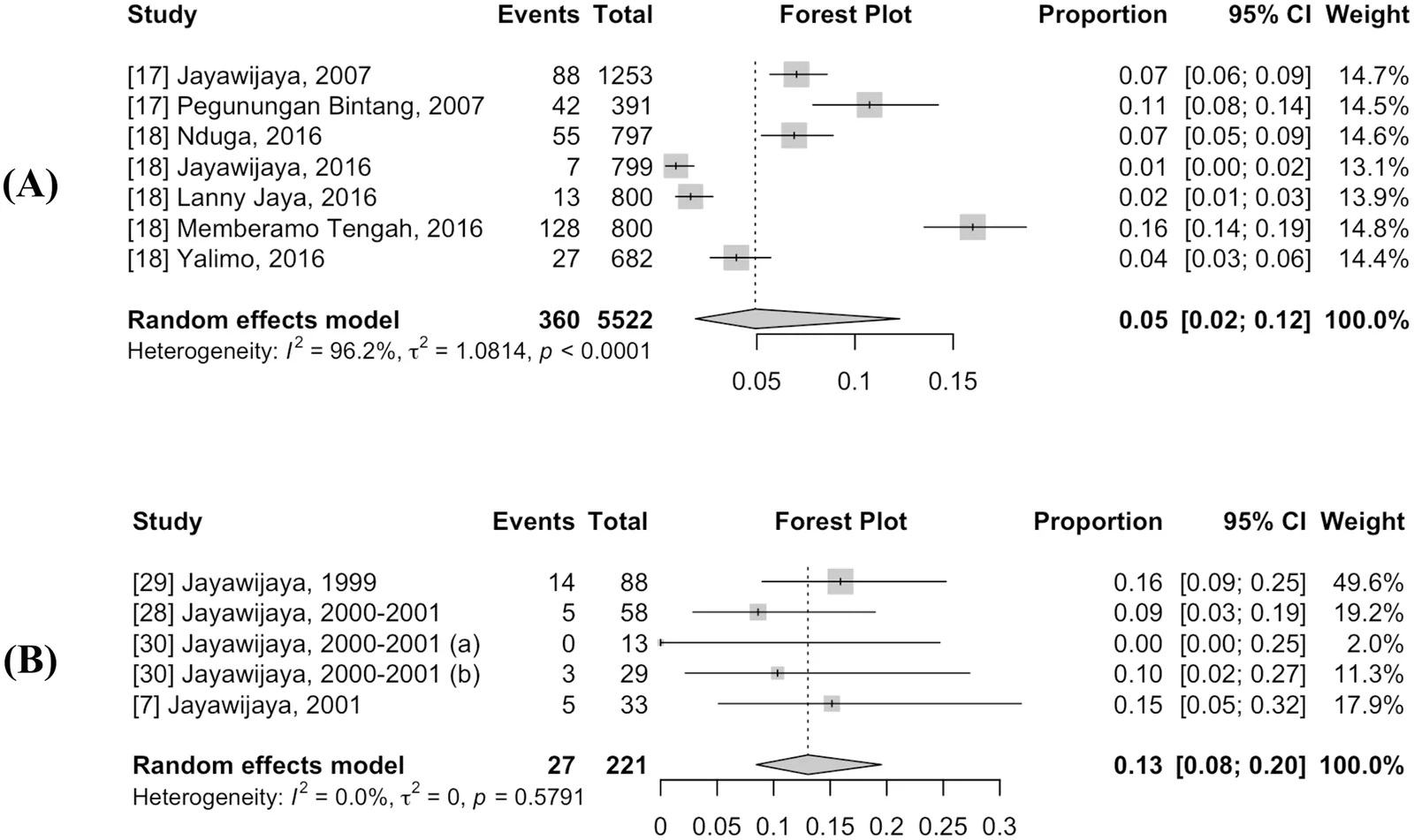
Pooled prevalence of *T. solium* taeniasis cases in Highland Papua. The forest plots represent taeniasis prevalence from community-based studies in Highland Papua using A) recombinant rES33 Enzyme-linked immunotransfer blot (EITB), and B) copro-Enzyme-linked immunosorbent assay (ELISA).

The pooled prevalence of *T. solium* taeniasis in Highland Papua (Fig 6), diagnosed with EITB, was 5.0% (95% CI: 2.0%–12.0%, I^2^: 96.2%) [17,18]. In contrast, studies using copro-antigen ELISA reported a pooled prevalence of 13.0% (95% CI: 8.0%–20.0%, I^2^: 0.0%) [7,29,30,39]. All studies using copro-antigen ELISA were from Jayawijaya Regency. Although copro-antigen ELISA studies showed relatively high prevalence, these studies generally had smaller sample sizes compared to those using EITB. This limitation was partly due to cultural barriers in early research phases, as residents were often reluctant to provide stool samples, leading to smaller study cohorts.

### *T. solium* cysticercosis seroprevalence in Papua, Indonesia (1970–2024)

**Fig 7:**
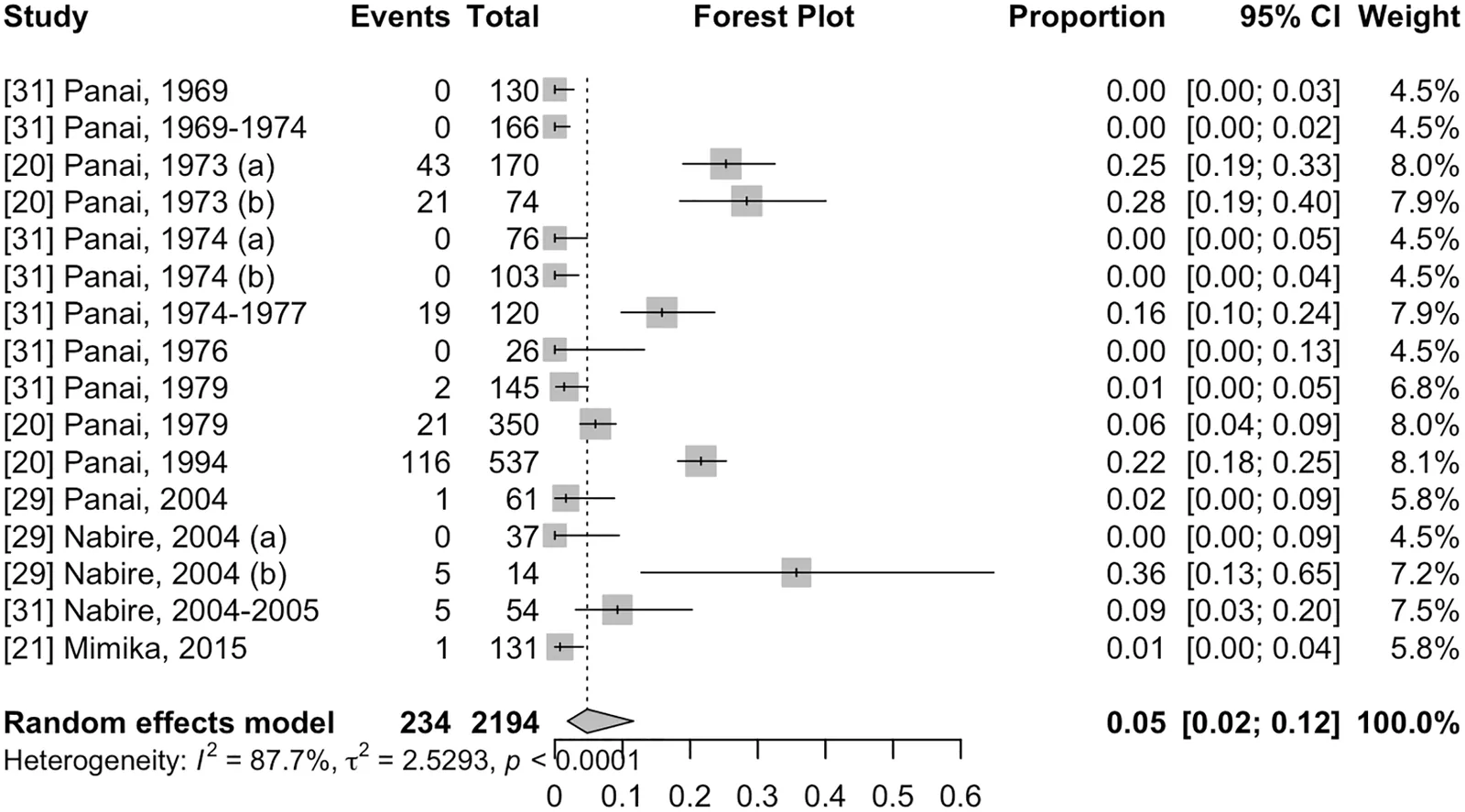
Pooled seroprevalence of *T. solium* cysticercosis in Central Papua using Enzyme-linked immunosorbent assay (ELISA).

Pooled seroprevalence estimates for *T. solium* cysticercosis in Papua were also produced when there were at least three study populations in a region evaluated using the same study design and diagnostic method. Pooled seroprevalence for NCC in Papua could not be determined, as only two articles described NCC cases, both from Central Papua. In Central Papua (Fig 7), the pooled community-based seroprevalence of cysticercosis using Ab-ELISA in asymptomatic populations was 5.0% (95% CI: 2.0%–13.0%, I^2^: 83.5%) [20,21,29,31]. Many of the earlier studies were conducted in the Paniai Regency of Central Papua. However, after 2002 research expanded to other areas of Central Papua, including the regencies of Nabire and Mimika.

**Fig 8:**
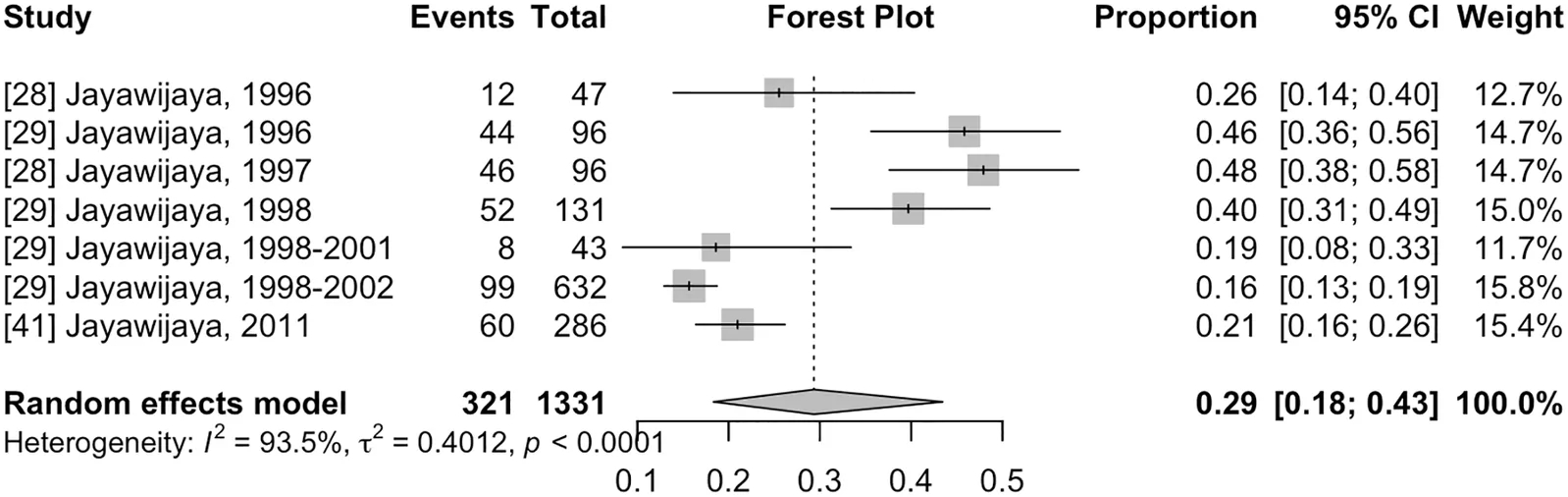
Pooled seroprevalence of *T. solium* cysticercosis in Highland Papua using antibody Enzyme-linked immunosorbent assay (Ab-ELISA).

In Highland Papua, the community-based cysticercosis pooled seroprevalence using ELISA in asymptomatic populations was 29.0% (95% CI: 18.0%–43.0%, I^2^: 93.5%) (Fig 8). This figure was derived from studies in Jayawijaya Regency conducted between 1996 and 2011 [28,29,41]. The highest seroprevalence occurred in a 1996 study and included 96 individuals from villages in the Assologaima sub-district of Jayawijaya, resulting in a seroprevalence of 48.0% (95% CI: 38.0%–58.0%) [28].

**Fig 9:**
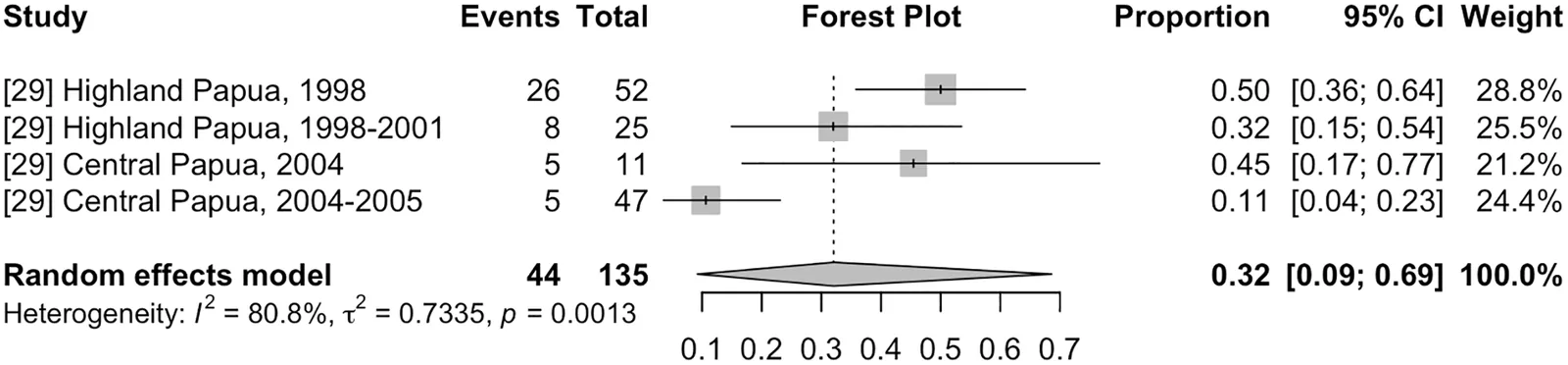
Proportion of individuals with headache and/or seizure among those seropositive for *T. solium* cysticercosis based on Ab-ELISA in community-based studies in Papua.

Ten studies evaluated cysticercosis seroprevalence in high-risk sub-populations. A study conducted between 1991 and 1995 using immunoblotting in Jayawijaya reported a cysticercosis seroprevalence of 66.67% (12/18) among residents with epileptic seizures and/or headaches, and 64.52% (20/31) among those with subcutaneous nodules [36]. Between 1998 and 1999, Margono et al. (2006) evaluated residents with epileptic seizures, subcutaneous nodules, and/or taeniasis in the Wamena Kota and Assologaima districts of Jayawijaya Regency. Using ELISA and/or immunoblotting, they reported positive proportions ranging from 19.51% (8/41) among individuals with epileptic seizures to 100% (12/12) in residents presenting with both seizures and subcutaneous nodules. Among individuals with taeniasis, 37.5% (6/16) were seropositive for cysticercosis [7]. Another study conducted in 1999 among residents with taeniasis found a seroprevalence of 50.0% (7/14) using ELISA [19]. Between 1998 and 2005, four community-based studies from Highland Papua and Central Papua examined the proportion of individuals with headache and/or seizure among those seropositive for *T. solium* cysticercosis based on Ab-ELISA (Fig 9). Across 135 patients, the pooled seroprevalence of residents experiencing headache and/or seizure were 32.0% (95% CI: 9.0%–69.0%, I^2^: 80.8%) [29].

### Clinical manifestations in individuals seropositive for cysticercosis by ELISA in Highland Papua (1970–2024)

**Fig 10:**
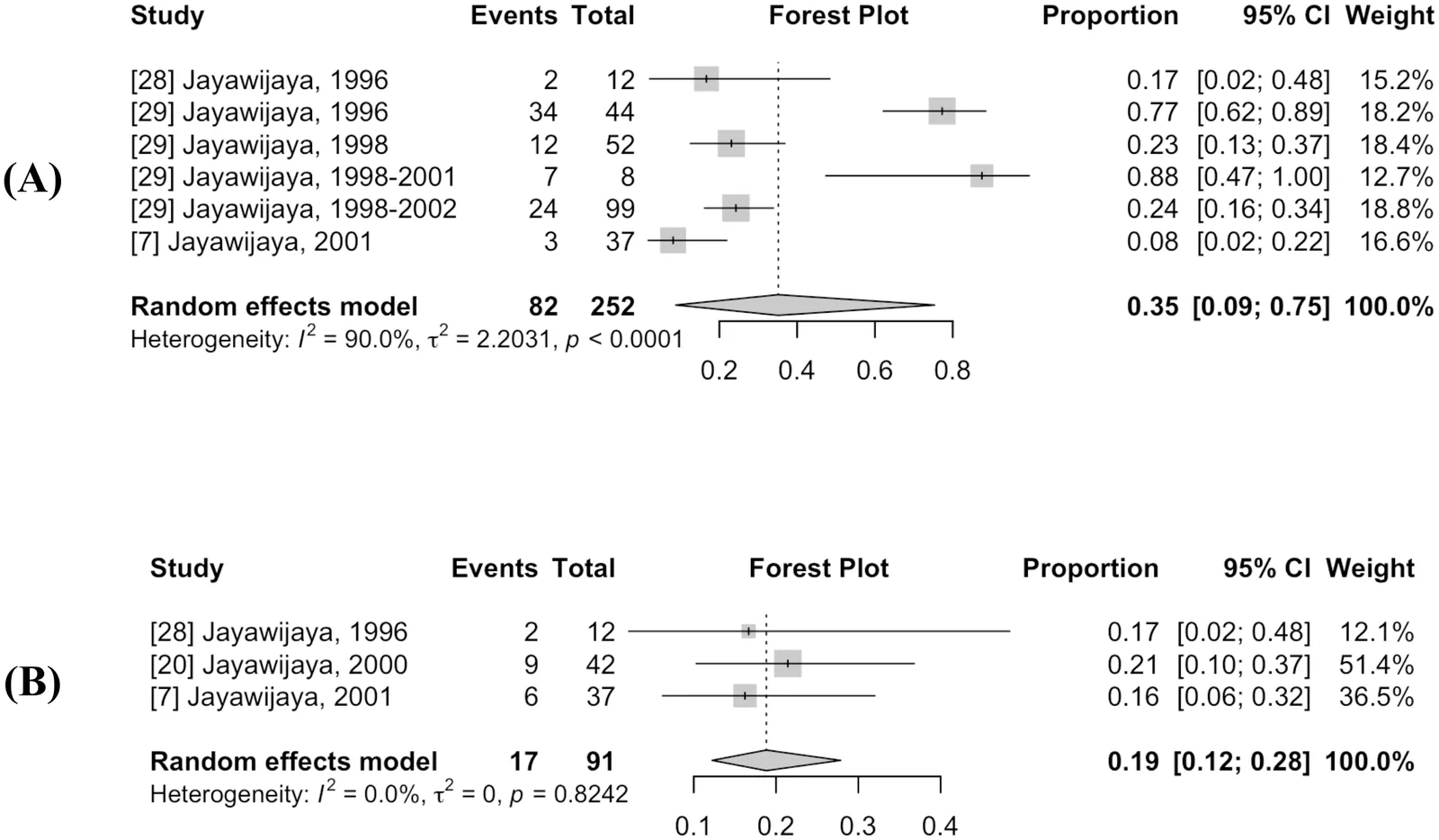
Pooled prevalence of clinical manifestations ((A) subcutaneous nodules and (B) seizures) among people positive by ELISA for *T. solium* cysticercosis in community-based studies in Papua.

Four articles reported community-based studies in Highland Papua that examined the distribution of clinical manifestations among individuals testing positive for *T. solium* cysticercosis using ELISA for detection of *T. solium* larvae IgG antibodies. Subcutaneous cysts were the most frequently reported clinical manifestation, with a pooled prevalence of 35.0% (95% CI: 9.0%–75.0%; I^2^:90.0%), while seizures had a pooled prevalence of 19.0% (95% CI: 12.0%–28.0%; I^2^: 0.0%) [7,20,28,29] (Fig 10).

### *T. solium* transmission risk areas in Papua (1970–2024)

**Fig 11:**
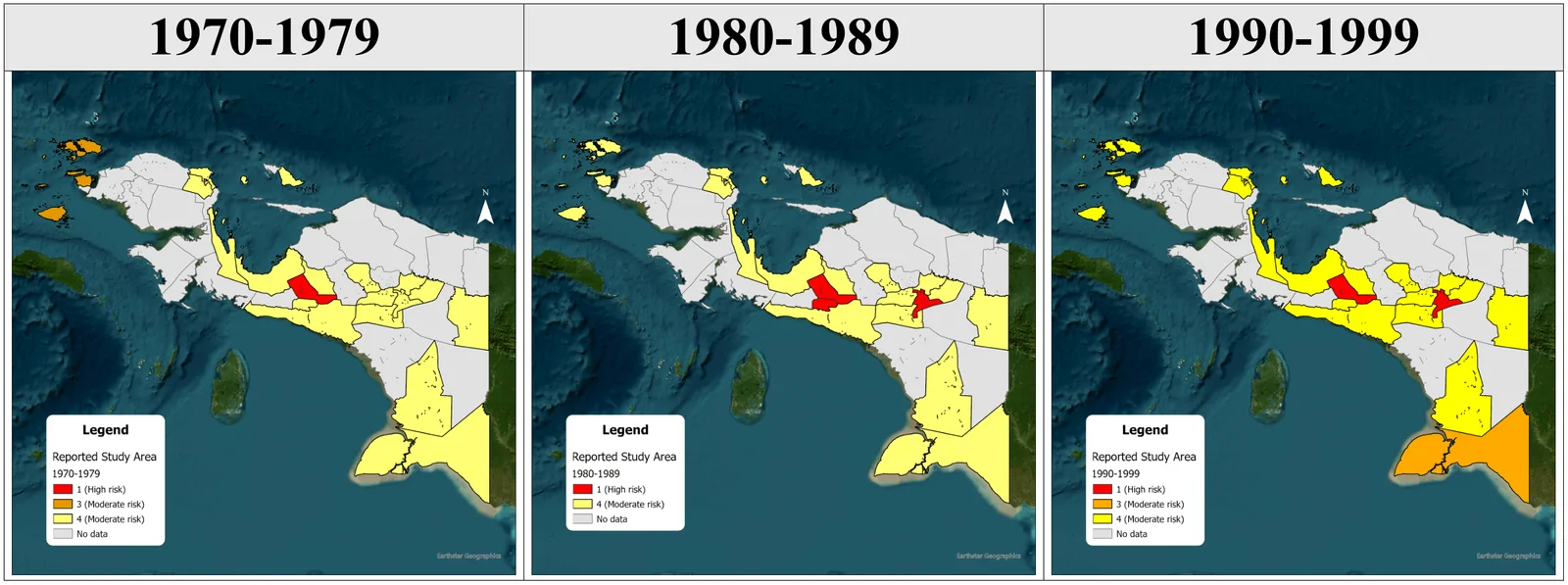
*Taenia solium* transmission risk areas in Papua based on the WHO mapping tool (1970–1999). Basemap tiles by Esri. Data sources: Esri, DigitalGlobe, GeoEye, i-cubed, USDA FSA, USGS, AEX, Getmapping, Aerogrid, IGN, IGP, swisstopo, and the GIS User Community (https://services.arcgisonline.com/ArcGIS/rest/services/World_Imagery/MapServer).

During the period from 1970 to 1979, most of Papua was classified as Risk Level 4 (moderate risk), except for Paniai in Central Papua, which was classified as Risk Level 1 due to the presence of reported taeniasis cases confirmed by stool examination and/or serology, cysticercosis cases identified through serological testing, and NCC cases confirmed by autopsy. In the same period, Raja Ampat in West Papua was categorized as Risk Level 3, reflecting reported cases of cysticercosis detected by ELISA and immunoblotting. From 1980 to 1989, Risk Level 1 classification expanded to include Deiyai in Central Papua, which reported additional taeniasis cases, and Jayawijaya in Highland Papua, which also registered new taeniasis cases. Other regions in Papua during this decade remained at moderate risk. From 1990 to 1999, Paniai in Central Papua and Jayawijaya in Highland Papua both maintained their Risk Level 1 classification, while Merauke in South Papua was designated as Risk Level 3 following reports of individuals seropositive for cysticercosis (Fig 11).

**Fig 12:**
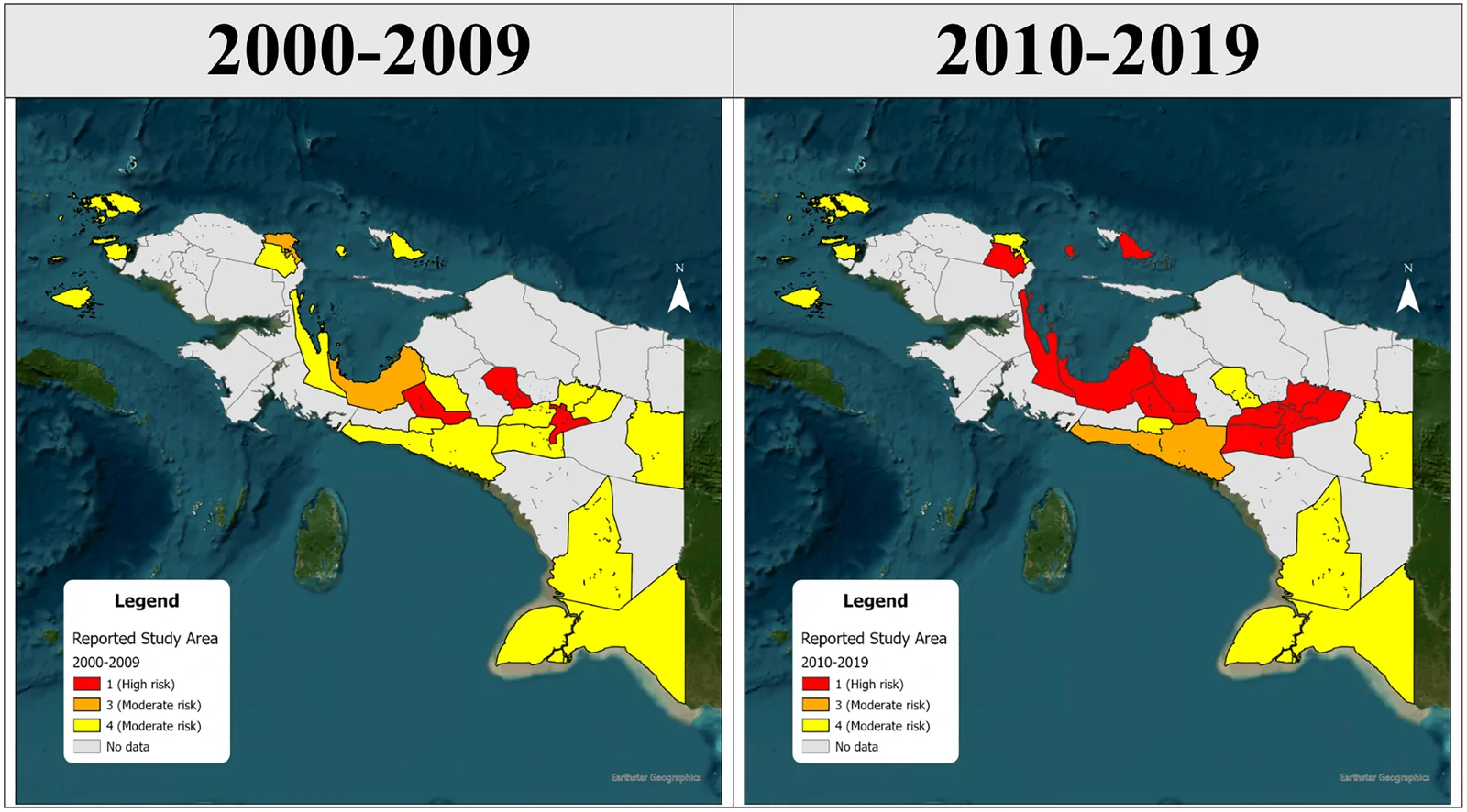
*Taenia solium* transmission risk areas in Papua based on the WHO mapping tool (2000–2019). Basemap tiles by Esri. Data sources: Esri, DigitalGlobe, GeoEye, i-cubed, USDA FSA, USGS, AEX, Getmapping, Aerogrid, IGN, IGP, swisstopo, and the GIS User Community (https://services.arcgisonline.com/ArcGIS/rest/services/World_Imagery/MapServer).

From 2000 to 2009, Risk Level 1 regions included Paniai, Jayawijaya, and Puncak Jaya in Central Papua. Puncak Jaya received its first Risk Level 1 classification due to newly reported cases of taeniasis supported by fecal testing and cysticercosis by serological testing. Meanwhile, Merauke in South Papua and Nabire in Central Papua were classified as Risk Level 3. During the last decade evaluated (2010–2019), there was a marked increase in regions classified as Risk Level 1 (Fig 12). Paniai and Jayawijaya remained classified at the highest risk level and were joined by Nabire and Intan Jaya in Central Papua; Nduga, Lanny Jaya, Mamberamo Tengah, and Yalimo in Highland Papua; Biak Numfor in Papua; and Teluk Wondama and Pegunungan Arfak in West Papua. The designation of these new high-risk zones was driven primarily by newly documented taeniasis cases. Despite a confirmed cluster of NCC cases in Mimika, Central Papua, in 2015 [21], this region was classified as Risk Area 3 due to the absence of reported taeniasis cases, which are essential to show active transmission potential. Transmission risk for 2020–present was not included in this analysis, as the estimations were based on the 28 articles identified in the scoping review, with the most recent data collected in 2016.

## Discussion

This scoping review represents the first attempt to synthesize over 50 years of data on *T. solium* taeniasis and cysticercosis/NCC in Papua, Indonesia, while also examining clinical manifestations among affected individuals. Globally, *T. solium* ranks highest in disability-adjusted life years (DALYs) among helminth infections of public health concern [42]. Yet, in Papua, most studies were conducted by independent researchers in highly localized areas, with no standardized surveillance system in place. In fact, a systematic review by Saelens and Gabriel (2020) identified only three articles describing existing surveillance programs worldwide [43–46], none of which were in Indonesia. Although *T. solium* was added to the WHO list of NTDs in 2010 and is included in the WHO NTD Road Map 2021–2030, studies in Papua demonstrate inconsistencies in methodology and frequent misalignment with WHO’s monitoring and evaluation framework [47]. These research challenges are compounded by long-standing and continuing armed conflict in Papua, where violence between Indonesian security forces and the West Papua National Liberation Army has disrupted civilian life and severely restricted humanitarian and research access, particularly since 2018 [48].

The geographic distribution of the 28 included studies shows a prominent concentration of cases in Central and Highland Papua. Paniai Regency in Central Papua emerged as the epicenter of taeniasis research focus, with 15 articles published between 1969 and 2007. Much of this early research centered on Enarotali Hospital, partly due to a burn epidemic among the Ekari people living around the Wissel Lakes. At that time, open-fire cooking practices were common, and frequent accidental falls into these fires during NCC-related epileptic episodes led to a high number of severe burn injuries [27]. However, this concentration of data likely reflects better hospital and research access rather than necessarily higher disease risk. South Papua and West Papua, remain underrepresented, with only two articles identified [22,29]. This geographic bias likely contributes to underestimation of the true burden of *T. solium* across the island. The apparent increase in higher-risk areas over time should also be interpreted with caution, as it may reflect improvements in surveillance, diagnostic capacity, and geographic coverage of studies and published case detection for *T. solium* taeniasis and human cysticercosis/NCC across Papua rather than a true increase in transmission.

Diagnostic methodology has also played a critical role in shaping reported prevalence values. In Central Papua, taeniasis pooled prevalence was estimated at 4.0% by microscopic stool analysis and 2.0% by EITB. Early studies relied on microscopy-based techniques such as egg and proglottid detection, Lugol’s iodine staining, and the Kato-Katz technique [21]. However, diagnosis via microscopy is limited by operator skill and parasite shedding patterns, leading to probable underestimation. EITB results may underestimate or overestimate prevalence if test sensitivity and specificity were not properly validated in Papua, where no published sensitivity/specificity assessments exist.

In Highland Papua, cysticercosis pooled seroprevalence in asymptomatic populations was estimated at 29.0% using Ab-ELISA. However, studies conducted in areas with known endemicity may be affected by sampling bias, especially when high-risk groups are preferentially tested. For example, a 1999 study in Highland Papua reported an exceptionally high prevalence of 63.16% using an Ab-ELISA test. However, the study population was derived from pig owners and their relatives or neighbours with known taeniasis, a population already at elevated risk of exposure [19,38]. Consequently, this sampling strategy likely overestimated prevalence when compared to what would be expected in the general population. A recent study confirmed these limitations. Hossain et al. (2025) showed that sensitivity of serodiagnostic tests for cysticercosis has not improved over time and is highly variable, often less than 30%, while specificity is generally higher [49]. In Brazil, Moraes et al. (2023) found high seroprevalence using Ab-ELISA, attributed to cross-reactivity and prior exposure rather than active infection [50].

Other tests offer better performance but still have important limitations. Ag-ELISA is highly sensitive and specific for patients with multiple viable cysts and is economical and reproducible [51]. However, this test performed poorly in patients with single viable cysts, where up to 35% of cases were false negatives [51]. Although the assay shows low cross-reactivity with other parasites, its diagnostic accuracy still depends on parasite burden. Currently, the best serological test to identify cysticercosis is the EITB. This test has a specificity approaching 100% and a sensitivity of 98% in individuals with two or more viable or degenerating cysts [49]. Yet, a positive EITB does not confirm CNS infection, and background seropositivity of 5–20% is common in endemic regions. EITB sensitivity drops substantially in patients with single intracranial cysts or calcified lesions, where up to 50% may be falsely negative [52]. Neither EITB nor Ag-ELISA are field-friendly, widely available, or available in point-of-care formats [53]. Given these constraints, neuroimaging remains essential. According to the United States Centers for Disease Control and Prevention (CDC) (2025), CT scans remain the gold standard for NCC diagnosis, as they can detect calcifications, edema, and cyst degeneration [54]. Reliance on serology alone risks overestimation, particularly in highly endemic areas with repeated sampling.

Diagnostic methods were also broadly defined across studies, ranging from ELISA to histopathology, creating further risk of heterogeneous data with varying levels of reliability [55]. Although, particular care was taken to ensure that the studies included in pooled prevalence estimates were methodologically and contextually comparable. Specifically, we restricted pooling to studies conducted within the same province, among the same type of population (e.g., general population, hospital-based patients, or targeted high-risk groups), and using the same diagnostic method. However, there was substantial heterogeneity across studies (I² ≥ 90.0%), even using a random-effects model. This variability likely reflects methodological inconsistencies, long study periods, small sample sizes, and the potential for repeated sampling within the same populations. In addition, confidence intervals were extremely wide for several pooled prevalence estimates, driven by limited sample sizes and high heterogeneity [55,56].

Evidence for NCC in Papua remains even more limited. Only two studies reported NCC cases, with just one employing CT imaging, which did not apply the Del Brutto diagnostic criteria [52]. In addition to the two included studies, Gajdusek (1977) described an autopsy of a 13-year-old Ekari girl from Central Papua who experienced seizures and burns before dying after being in a coma [23]. Ultimately, this study was excluded from this review due to the absence of a study date. Some studies incorrectly used the term NCC based solely on serology, although serological tests cannot confirm cyst(s) localization to the brain. According to Del Brutto, definitive NCC diagnosis requires neuroimaging criteria paired with clinical and serological evidence, yet these standards were rarely met in Papua [52]. As a result, the true NCC burden is almost certainly underestimated. Therefore, global estimates of *T. solium* burden often exclude Indonesia due to insufficient diagnostic rigor and poor-quality data.

Open defecation and backyard pig rearing sustain the life cycle of *T. solium* in Papua. Cultural practices such as *bakar batu*, a traditional feast involving pork cooked with hot stones, pose additional risks when meat is undercooked. According to the WHO (2022), 4.19% of Indonesia’s population practices open defecation, with rural areas (6.96%) more affected than urban settings (2.18%) [9]. Combined with limited surveillance, poor hygiene in food preparation, and inadequate meat inspection systems, these practices perpetuate *T. solium* transmission. Studies also note a high epilepsy burden in Papua, which may be linked to undiagnosed NCC [57]. The Indonesian health system has yet to integrate *T. solium* into a One Health framework, despite the persistence of transmission for over 50 years. Our risk mapping analysis shows active transmission. Armed conflict and humanitarian crises further compromise healthcare access, with 87% of internally displaced persons from Papua reporting limited or no healthcare access in 2024 [48,58]. This crisis likely worsens food insecurity, hygiene conditions, and surveillance gaps, sustaining the *T. solium* life cycle and transmission risk.

Evaluation of porcine cysticercosis data was outside the scope of this review, therefore, risk classification based on the WHO framework, which incorporates both human and porcine data, may be incomplete within the context of this analysis. Since apparent prevalence, rather than “true” or “informed” prevalence, is reported due to lack of test sensitivity and specificity information, all pooled prevalence values should be interpreted cautiously. This limitation should be considered when comparing infection burden across studies [16].

The mapping and evidence landscaping approach employed in this scoping review provides a critical historical synthesis of *T. solium* in Papua; however, it differs substantially from recent high-resolution geospatial and modelling initiatives applied in other endemic settings, including Laos PDR, Malawi, Uganda, and Colombia, where spatial autocorrelation, geostatistical modelling, and integrated surveillance data have been used to generate fine-scale risk maps and predictive surfaces [59–64]. By design, this study adopts a scoping review framework to prioritize the mapping of existing evidence and the identification of methodological heterogeneity across five decades of literature (1970–2024), rather than performing formal prevalence adjustment or predictive spatial modelling.

While these approaches enable formal statistical inference and high-resolution risk prediction, their application depends on the availability of standardized surveillance systems, consistent diagnostic reporting, and sufficient spatially resolved data [11]. In the context of Papua, such requirements are not currently met due to long-standing gaps in surveillance infrastructure, heterogeneous diagnostic methodologies, and the absence of systematically collected animal health data, which further limits the ability to fully characterize transmission dynamics. Therefore, a scoping and descriptive mapping approach was necessary to synthesize fragmented evidence and characterize the distribution of available studies. Together, these findings establish an essential baseline for Papua and clearly demonstrate that advanced geospatial and predictive risk mapping will require improved diagnostic standardization and integrated surveillance data [11].

The substantial disease burden in Central and Highland Papua calls for targeted interventions, including standardized surveillance. Use of the 2024 WHO *T. solium* control, monitoring, and evaluation framework is recommended to ensure consistent surveillance [47]. Such efforts, when combined with improved pig husbandry practices and measures to reduce exposure of pigs to human fecal contamination, have the potential to interrupt the parasite life cycle. In parallel, access to appropriate diagnostic tools, including neuroimaging modalities such as CT and magnetic resonance imaging (MRI), should be expanded through strengthened referral systems and improved diagnostic capacity at regional health facilities. NCC diagnosis should strictly apply the Del Brutto criteria to improve reliability [52]. Public health programs focusing on hygiene education and safe cooking practices are critical, particularly in Indigenous communities. Community- and school-based screening strategies may offer opportunities for early detection and awareness-raising in high-risk populations. Future studies should prioritize longitudinal studies and increased research efforts are needed in underrepresented areas. Investigating socio-economic, cultural, and environmental factors driving regional differences will provide valuable insights for targeted public health interventions.

## Conclusions

This study reviewed existing literature on the frequency and clinical characteristics of *T. solium* taeniasis and cysticercosis/NCC in Papua, Indonesia, from 1970 to 2024. While the findings are concerning, the true burden of *T. solium* infections remains unclear due to limitations in diagnostic methods and data availability. To address these neglected parasitic diseases, there is a critical need for improved diagnostic tools (e.g., CT scans), more comprehensive research, and targeted public health interventions, especially for Indigenous communities with limited access to education and healthcare.

## Supporting information

S1 Checklist Preferred Reporting Items for Systematic reviews and Meta-Analyses extension for Scoping Reviews (PRISMA-ScR) Checklist. (PDF)

S1 Table Binary Classification of *Taenia solium* Transmission Risk^a^ Areas in Papua (1970–2025). (PDF)
