# Supplementary material for: A scoping review of the frequency and clinical presentation of Taenia solium taeniasis and human cysticercosis/neurocysticercosis in Papua, Indonesia from 1970 to 2024
Source: PLoS Negl Trop Dis. 2026 Sep 8;20(9):e0014702. doi: 10.1371/journal.pntd.0014702 (PMC13588522; doi:10.1371/journal.pntd.0014702)
Supplement: Supplementary file 2 [file pntd.0014702.s002.pdf]

S1 Table. Binary Classification of *Taenia solium* Transmission Risk[a] Areas in Papua (1970–2025) Based on the WHO Mapping Protocol and Presence of Reported Positive Cases of Taeniasis and Human Cysticercosis/NCC Using Acceptable Diagnostic Methods[b].

Regency	Province	1970-1979			Risk	1980-1989			Risk	1990-1999			Risk	2000-2009			Risk	2010-2019			Risk
		T	C	NCC		T	C	NCC		T	C	NCC		T	C	NCC		T	C	NCC	
Paniai	Central Papua	YES	YES	YES	1	YES	YES	NO	1	YES	YES	NO	1	YES	YES	NO	1	YES	YES	NO	1
Deiyai	Central Papua	NO	NO	NO	4	YES	NO	NO	1	NO	NO	NO	4	NO	NO	NO	4	NO	NO	NO	4
Nabire	Central Papua	NO	NO	NO	4	NO	NO	NO	4	NO	NO	NO	4	NO	YES	NO	3	YES	NO	NO	1
Puncak Jaya	Central Papua	NO	NO	NO	4	NO	NO	NO	4	NO	NO	NO	4	YES	YES	NO	1	NO	NO	NO	4
Mimika	Central Papua	NO	NO	NO	4	NO	NO	NO	4	NO	NO	NO	4	NO	NO	NO	4	NO	YES	YES	3
Intan Jaya	Central Papua	NO	NO	NO	4	NO	NO	NO	4	NO	NO	NO	4	NO	NO	NO	4	YES	NO	NO	1
Pegunungan Bintang	Highland Papua	NO	NO	NO	4	NO	NO	NO	4	NO	NO	NO	4	YES	YES	NO	4	NO	NO	NO	4
Jayawijaya	Highland Papua	NO	NO	NO	4	YES	NO	NO	1	YES	YES	NO	1	YES	YES	NO	1	YES	NO	NO	1
Nduga	Highland Papua	NO	NO	NO	4	NO	NO	NO	4	NO	NO	NO	4	NO	NO	NO	4	YES	NO	NO	1
Lanny Jaya	Highland Papua	NO	NO	NO	4	NO	NO	NO	4	NO	NO	NO	4	NO	NO	NO	4	YES	NO	NO	1
Memberamo Tengah	Highland Papua	NO	NO	NO	4	NO	NO	NO	4	NO	NO	NO	4	NO	NO	NO	4	YES	NO	NO	1
Yalimo	Highland Papua	NO	NO	NO	4	NO	NO	NO	4	NO	NO	NO	4	NO	NO	NO	4	YES	NO	NO	1
Mapia	Papua	NO	NO	NO	4	NO	NO	NO	4	NO	NO	NO	4	NO	NO	NO	4	NO	NO	NO	4
Biak Numfor	Papua	NO	NO	NO	4	NO	NO	NO	4	NO	NO	NO	4	NO	NO	NO	4	YES	NO	NO	1
Merauke	South Papua	NO	NO	NO	4	NO	NO	NO	4	NO	YES	NO	3	NO	NO	NO	4	NO	NO	NO	4
Raja Ampat	West Papua	NO	YES	NO	3	NO	NO	NO	4	NO	NO	NO	4	NO	NO	NO	4	NO	NO	NO	4
Manokwari	West Papua	NO	NO	NO	4	NO	NO	NO	4	NO	NO	NO	4	NO	YES	NO	3	NO	NO	NO	4
ondama	West Papua	NO	NO	NO	4	NO	NO	NO	4	NO	NO	NO	4	NO	NO	NO	4	YES	YES	NO	1
Pegunungan Arfak	West Papua	NO	NO	NO	4	NO	NO	NO	4	NO	NO	NO	4	NO	NO	NO	4	YES	YES	NO	1

[a]: Risk Area Classification:

Risk Areas 1 and 2 (High Risk): Evidence or strong likelihood of active transmission of *T. solium*.

Risk Areas 3 and 4 (Moderate Risk): Indications of/or potential for active transmission.

Risk Areas 5 and 6 (Lower Risk): Uncertain or limited evidence; active transmission may or may not be present.

The classification is derived using a binary Yes/No system based on the presence or absence of positive *T. solium* taeniasis and human cysticercosis/NCC cases detected with acceptable diagnostic methods, as defined by WHO:

- **YES**: Indicates that positive cases or risk factors have been recently confirmed using acceptable diagnostic techniques.
- **NO**: Indicates no evidence of recent positive cases or risk factors using eligible methods.
- **UNKNOWN**: Indicates insufficient or unavailable data.

Disease				Risk factors		Risk level
Porcine cysticercosis	Taeniasis by *T. solium*	Taeniasis	Neuro-cysticercosis	Open defecation	Backyard pigs common	
Unknown	Yes	Yes	Yes	Yes	Yes	**1**
Unknown	Yes	Yes	No	Yes	Yes	**1**
Unknown	Yes	Yes	Unknown	Yes	Yes	**1**
Unknown	Yes	No	Yes	Yes	Yes	**1**
Unknown	Yes	No	No	Yes	Yes	**1**
Unknown	Yes	No	Unknown	Yes	Yes	**1**
Unknown	Yes	Unknown	Yes	Yes	Yes	**1**
Unknown	Yes	Unknown	No	Yes	Yes	**1**
Unknown	Yes	Unknown	Unknown	Yes	Yes	**1**
Unknown	No	Yes	Yes	Yes	Yes	**2**
Unknown	No	Yes	No	Yes	Yes	**2**
Unknown	No	Yes	Unknown	Yes	Yes	**2**
Unknown	No	No	Yes	Yes	Yes	**3**
Unknown	No	No	No	Yes	Yes	**4**
Unknown	No	No	Unknown	Yes	Yes	**4**
Unknown	No	Unknown	Yes	Yes	Yes	**3**
Unknown	No	Unknown	No	Yes	Yes	**4**
Unknown	No	Unknown	Unknown	Yes	Yes	**4**
Unknown	Unknown	Yes	Yes	Yes	Yes	**2**
Unknown	Unknown	Yes	No	Yes	Yes	**2**
Unknown	Unknown	Yes	Unknown	Yes	Yes	**2**
Unknown	Unknown	No	Yes	Yes	Yes	**3**
Unknown	Unknown	No	No	Yes	Yes	**4**
Unknown	Unknown	No	Unknown	Yes	Yes	**4**
Unknown	Unknown	Unknown	Yes	Yes	Yes	**3**
Unknown	Unknown	Unknown	No	Yes	Yes	**4**
Unknown	Unknown	Unknown	Unknown	Yes	Yes	**NS***

*Insufficient information to classify risk

For the time period analyzed, the classification assumes the widespread presence of two key *T. solium* risk factors across Papua (backyard pig rearing is common and open defecation practices persist in many areas) (WHO, 2022).

[b]: Acceptable diagnostic methods include:

- **Taeniasis (T):** Microscopy (e.g., Kato-Katz), copro-antigen detection, serology (e.g., ELISA), or molecular techniques (e.g., PCR).
- **Human Cysticercosis (C):** Diagnosis through serological testing, neuroimaging (CT/MRI), or a combination of these methods.
- **Neurocysticercosis (NCC):** Typically diagnosed through imaging in referral hospitals or specialized centers. These diagnoses may not indicate where the infection was acquired. In areas where imaging is not widely available, **epilepsy is used as a proxy indicator** of potential NCC (WHO, 2023).

Reference:

1. WHO. WASH exposure, defecation: population practising open defecation (%) [Internet]. 2022. [cited 2025 Jun 30]. Available from: https://www.who.int/data/gho/data/indicators/indicator-details/GHO/population-practising-open-defecation-(-)
2. WHO. Mapping protocol for Taenia solium: identification of endemic and high-risk areas [Internet]. 2023. [cited 2025 Jun 30]. Available from: https://iris.who.int/handle/10665/372868. License: CC BY-NC-SA 3.0 IGO.
